# Effects of the EQUIP quasi-experimental study testing a collaborative quality improvement approach for maternal and newborn health care in Tanzania and Uganda

**DOI:** 10.1186/s13012-017-0604-x

**Published:** 2017-07-18

**Authors:** P Waiswa, F Manzi, G Mbaruku, A. K. Rowe, M Marx, G Tomson, T Marchant, B. A. Willey, J Schellenberg, S Peterson, C Hanson, The EQUIP study team, J. Akuze, P. Arafumin, U. Baker, H. Balidawa, J. Jaribu, D. Kajjo, J. Kalungi, B. Kawala, A. Majura, R. Mandu, I. Msonde, M. Okuga, D. Saulnier, Y. Sedekia, T. Tancred, S. Temu

**Affiliations:** 1 College of Health Sciences, School of Public Health, Makerere University, Kampala, Uganda; 2 Department of Public Health Sciences, Karolinska Institutet, Stockholm, Sweden; 3 Ifakara Health Institute, Dar-es-Salaam, Tanzania; 4 Malaria Branch, Division of Parasitic Diseases and Malaria, Center for Global Health, Centers for Disease Control and Prevention, Atlanta, GA USA; 5 Evaplan GmbH the University of Heidelberg, Heidelberg, Germany; 6 Department of Learning, Informatics, Management, Ethics, Karolinska Institutet, Stockholm, Sweden; 7 Department of Disease Control, London School of Hygiene and Tropical Medicine, London, UK; 8 Department Infectious Disease Epidemiology, London School of Hygiene and Tropical Medicine, London, UK; 9 International Maternal and Child Health Unit, Department of Women’s and Children’s Health, Uppsala University, Uppsala, Sweden

## Abstract

**Background:**

Quality improvement is a recommended strategy to improve implementation levels for evidence-based essential interventions, but experience of and evidence for its effects in low-resource settings are limited. We hypothesised that a systemic and collaborative quality improvement approach covering district, facility and community levels, supported by report cards generated through continuous household and health facility surveys, could improve the implementation levels and have a measurable population-level impact on coverage and quality of essential services.

**Methods:**

Collaborative quality improvement teams tested self-identified strategies (change ideas) to support the implementation of essential maternal and newborn interventions recommended by the World Health Organization. In Tanzania and Uganda, we used a plausibility design to compare the changes over time in one intervention district with those in a comparison district in each country. Evaluation included indicators of process, coverage and implementation practice analysed with a difference-of-differences and a time-series approach, using data from independent continuous household and health facility surveys from 2011 to 2014. Primary outcomes for both countries were birth in health facilities, breastfeeding within 1 h after birth, oxytocin administration after birth and knowledge of danger signs for mothers and babies. Interpretation of the results considered contextual factors.

**Results:**

The intervention was associated with improvements on one of four primary outcomes. We observed a 26-percentage-point increase (95% CI 25–28%) in the proportion of live births where mothers received uterotonics within 1 min after birth in the intervention compared to the comparison district in Tanzania and an 8-percentage-point increase (95% CI 6–9%) in Uganda. The other primary indicators showed no evidence of improvement. In Tanzania, we saw positive changes for two other outcomes reflecting locally identified improvement topics. The intervention was associated with an increase in preparation of clean birth kits for home deliveries (31 percentage points, 95% CI 2–60%) and an increase in health facility supervision by district staff (14 percentage points, 95% CI 0–28%).

**Conclusions:**

The systemic quality improvement approach was associated with improvements of only one of four primary outcomes, as well as two Tanzania-specific secondary outcomes. Reasons for the lack of effects included limited implementation strength as well a relatively short follow-up period in combination with a 1-year recall period for population-based estimates and a limited power of the study to detect changes smaller than 10 percentage points.

**Trial registration:**

Pan African Clinical Trials Registry: PACTR201311000681314

**Electronic supplementary material:**

The online version of this article (doi:10.1186/s13012-017-0604-x) contains supplementary material, which is available to authorized users.

## Background

In sub-Saharan Africa, maternal and newborn mortality remain unacceptably high. Over one million newborns and 201,000 pregnant or post-partum women died in 2015 in sub-Saharan Africa alone [1, 2], despite the wide promotion of effective and affordable interventions to prevent these deaths [3]. Implementation levels of essential evidence-based interventions for maternal and newborn health vary within and between countries [4] with major quality gaps. In Tanzania and Uganda, essential interventions such as the active management of the third stage of labour or measuring blood pressure during antenatal care should be implemented according to national guidelines, but actual coverage remains limited for several reasons including low availability of essential items in facilities [5–8], weak governance and substandard health care worker practices [9]. Quality management, including quality improvement (QI) approaches, have the potential to improve coverage by optimising use of existing resources rather than adding more resources. Quality management includes monitoring quality, changing processes to improve performance and using locally generated data to test changes in a structured approach based on plan-do-study-act (PDSA) cycles [10].

The collaborative approach to QI as developed by the Institute of Healthcare Improvement [11, 12], includes the use of a group of health facility teams, training or sensitization towards standards, coaching and mentoring and learning across teams. Evidence of the effectiveness of the approach is increasingly available for high-resource [13, 14] as well as low-resource settings where QI has improved implementation levels of essential interventions [15], the scale-up of new interventions [16–18] or strengthened the whole parts of the health system [19, 20]. Internal monitoring data produced by QI teams have been used in most of these assessments [15–20]. Few QI strategies have been evaluated using independent population- and facility-based data, with the MaiKhanda trial in Malawi being one example [21]. There is also little evidence on how QI can strengthen quality of care at facilities, or district health systems, when there is a lack of financial and human resources, drugs and supplies; thus, the effect of QI on strengthening health systems in low-resource settings is understudied [22]. In addition, QI has rarely been used concurrently at primary level health facilities and in communities [19], or indeed, at all levels of an entire district system.

The Expanded Quality Management Using Information Power (EQUIP) strategy was developed to increase the coverage and quality of essential interventions for maternal and newborn care in two high-mortality settings of Tanzania and Uganda [23] and was based on the increasing evidence that collaborative QI can improve implementation levels for essential interventions [15]. We included a community component because of the evidence of the effect of community programmes on newborn mortality [24, 25]. In contrast to many approaches, to evaluate QI interventions that use monitoring data collected by the implementing QI teams themselves [15, 18], EQUIP also aimed to generate evidence on the impact of QI at the population level by means of continuous household and health facility surveys covering QI intervention and comparison areas [26].

The EQUIP hypothesis was that QI implemented at the district, facility and community levels and supported by report cards, generated through continuous household and health facility surveys, could have a measurable population-level impact on demand for and supply of high-quality maternal and newborn health care services. By focusing on all levels of the health care system and by including continuous surveys to increase the availability of high-quality information, EQUIP was expected to have a health-system-strengthening effect [27]. This responded to the need to build inclusive, patient-centred health systems [28], with a continuum of care approach, from the community to the primary health facility and to the hospital care.

Here, we report the effect of the EQUIP intervention on the coverage and quality of essential maternal and newborn health care interventions and knowledge of danger signs after 15 and 26 months of full implementation in Uganda and Tanzania, respectively.

## Methods

### Study setting, trial design and participants

**Fig. 1:**
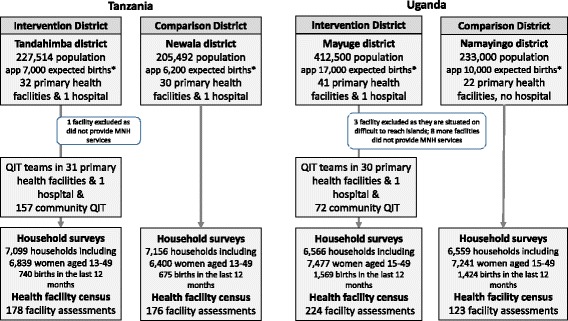
Trial design. (*asterisk*) Estimates per year using the birth rate observed by continuous household survey

We used a quasi-experimental, plausibility design to evaluate EQUIP [29] (Fig. 1) and compared two purposefully selected districts in each of Southern Tanzania and Eastern Uganda; a detailed description of the intervention and study design is presented elsewhere [23, 30]. Briefly, the two pairs of districts were selected from areas where the research team had established research collaborations and (1) the districts were rural, so results would be relevant to other rural districts in the two countries and (2) the districts were of comparable size with similar health infrastructure. However, in Uganda, the government split our comparison district 3 months after our decision was made in December 2010. Of the two new districts, we selected the newly created district Namayingo, as this was the most similar to the intervention district in terms of geographical features (both border Lake Victoria). This meant that the comparison district in Uganda lacked a hospital, had a smaller population than the intervention district and had a less developed health infrastructure (Table 1).

**Table 1:** Population and health system characteristics

	Tanzania		Uganda	
	Tandahimba (intervention district)	Newala (comparison district)	Mayuge (intervention district)	Namayingo (comparison district)
Population	227,514	205,492	412,500	233,000
Socio-economic characteristics of household				
Possession of mobile phone	48%	47%	70%	64%
Possession of tin/tile roof	53%	43%	74%	39%
House with electricity	3%	1%	3%	1%
Financing per capita spent on health per year, according to district reports	7 USD	12 USD	6 USD^a^	
Governance and leadership	Good continuity, some vision, increasingly bottom-up planning, good collaboration with partners	Interruption in leadership, clear vision, strong team spirit, bottom-up planning and good collaboration with partners	Interruption in leadership, clear vision, good team spirit, bottom-up planning	New team, some involvement of communities,
Human resources				
% of posts filled	39%	43%	61%	47%
In-service training courses	Family planning, HIV, PMTCT and district management 1 course in emergency obstetric care	Family planning, HIV, PMTCT and district management	Life-saving skills, Helping Babies Breathe (HBB) and Kangaroo Mother Care	Not assessed
Total number of nurses per 1000 population	0.97	0.79	0.70	0.53
Total number of prescribers per 1000 population	0.51	0.31	0.10	0.10
Drugs and supplies at facilities^b^	1st round/6th round	1st round/6th round	1st round/6th round	1st round/6th round
Oxytocin	39%/93%	56%/90%	24%/57%	9%/25%
Syphilis test	67%/18%	54%/10%	56%/33%	63%/33%
Injectable ampicillin	9%/10%	0%/6%	21%/17%	5%/15%
MG-sulphate	21%/30%	70%/46%	3%/0%	0%/0%
Clamp/umbilical ties	78%/97%	64%/82%	29%/57%	9%/65%
Resuscitation device/Ambu bag	30%/100%	48%/55%	32/43%	18%/65%
Health information use for planning	HMIS, no other sources	HMIS, no other sources	HMIS EQUIP data and other survey information used	
Delivery system infrastructure				
Hospital/primary facilities	1 hospital, 33 primary facilities (1 private)	1 hospital, 29 primary facilities (no private)	1 hospital, 41 primary facilities (8 private without MNC-services)	No hospital, 20 primary facilities
Basic infrastructure^c^				
Electricity available	57%	43%	32%	10%
Running water available	72%	96%	54%	46%
Referral Ambulances/referral system	1 ambulance: formal referral system established shortly before the end of project	2 ambulances/formal referral system established	1 ambulance in poor condition	1 ambulance in poor condition
Phone/communication with referral facility for last referral	41%	49%	18%	25%

^a^Windisch et al. National and district expenditure, p112 (ref [43])

^b^Information presents availability of the respective equipment and supply at the day of the health facility survey. 1st round of health facility survey took place from November 2011 to February 2012 and the sixth round from January to April 2014

^c^Relates to an average spanning over the six rounds of data collection as no variation was observed

The intervention districts of Tandahimba and Mayuge mainland and the two comparison districts of Newala and Namayingo, in Tanzania and Uganda, respectively, were predominantly rural. In Tandahimba, Tanzania, 32 public and faith-based health facilities offered maternal and newborn care for a population of 227,514. In Mayuge, Uganda, 33 public and faith-based facilities served a population of 412,500 (a ratio of 1.4 and 0.8 facilities, respectively, per 10,000 population). We did not include private for-profit facilities, as the few facilities operating in the study areas did not provide maternal and childbirth services (Additional file 1: webannex 1 maps).

In each country, we compared time trends of coverage and health care quality indicators and mothers’ knowledge of danger signs between intervention and comparison districts using independent continuous facility and household surveys. The plausibility design also included the regular assessment of contextual factors likely to affect maternal and newborn health other than the study intervention, as recommended by Victora et al. [31]. Community members and health staff were not masked to the intervention. The survey team worked independently and was trained to be as neutral as possible but was not masked to the intervention area. However, the survey team was unaware of the primary and secondary outcomes that were chosen for evaluation.

### Intervention

We based the QI approach on the collaborative model of improvement [11], which is a short-term, rapid-learning method to improve quality in a focused area based on PDSA cycles and clearly defined and agreed upon indicators for monitoring [32]. We described our methodology in more details in our protocol paper and in the annexes of our protocol paper [23, 33].

In intervention districts, we implemented two strategy components (1) collaborative QI with (a) district health managers, (b) health facility staff and (c) community health workers and (2) continuous household and health facility surveys, with results communicated to district health managers, health facilities and communities using report cards once every 4 months [26]. In comparison districts, we implemented continuous household and health facility surveys for evaluation, with results communicated to district health managers using a written report once per year [30].

For the QI strategy, the main health facility participants were health staff working in the area of maternal and newborn health. Community participants were volunteers, either selected by the community, often on grounds of prior experience as community volunteers (Tanzania) or chosen from active village health teams (in Uganda). The district QI team was composed of the council health management team (Tanzania) and the district health team (Uganda).

Every 3 to 4 months, we invited both health facility and community members to participate in learning sessions to review progress. Learning sessions were either joint or separate depending on the chosen improvement topic. These sessions, which typically lasted 1 day, introduced and reminded participants about QI techniques, including the PDSA cycles, and new topics for improvement and also provided a platform to review progress and allow teams to learn from each other. Separate learning sessions were held with district managers (Additional file 1: webannex 2 table). During action periods, which were times between learning sessions when the teams implemented the improvement work, the QI teams were mentored regularly by EQUIP project staff and district managers. In Tanzania, health facility and community QI teams were mentored on average two to three times each quarter, half of the time in the form of “cluster meetings”, where health facility and community QI teams met together locally. Similarly, in Uganda, two to three coaching and mentoring visits for health facility and community QI teams were done per quarter. The learning sessions were supported with feedback from the survey results presented in the form of report cards covering selected indicators (Additional file 1: webannex 5 report cards). The EQUIP team met with district health managers 11 and 12 times over the 30 months of EQUIP implementation in Tanzania and Uganda, respectively.

A piloting period took place in both intervention districts from June to October 2011. In Tanzania, full implementation, after the pilot and roll-out periods between June 2011 and February 2012, was achieved in 31 primary health facilities, one hospital and 157 villages (including each roughly 250–500 households each) from March 2012 to April 2014. In Uganda, gradual implementation was done from November 2011 to February 2013, and QI teams were active in 31 health facilities and 72 parishes (each comprising almost 1000 households) from February 2013 to April 2014 (Additional file 1: webannex 3 timeline of assessment and implementation).

QI teams selected improvement topics from a QI charter, a planning tool where key areas where improvement is needed are outlined (Additional file 1: webannex 4, improvement charter) which used the WHO guideline of recommended essential interventions, commodities and guidelines as a basis [3]. During the course of the project, prioritisation of which essential intervention to address first were frequently chosen based on discussions between project staff and district mentors and taking into account continuous survey results, which were summarised in report cards. QI teams implemented and tested various change ideas, such as new strategies for birth preparation counselling (e.g. going through birth preparation checklists) or changing implementation routines to address defined problems (e.g. having a delivery tray including oxytocin prepared at all times, see more details Additional file 1: webannex 6 vignettes). The community QI teams implemented a variety of change ideas (Table 2), including home visits to pregnant women, community discussions and the establishment of community savings funds. The improvement topics ‘facility delivery’, ‘uterotonics within one minute’ and ‘post-partum care’ were introduced early on in both countries. In Tanzania, the 1-day training, ‘Helping Babies Breathe’ was offered in March 2013 during one learning session for all intervention facilities preceding the national roll-out in the region. Health facility QI teams used job aids, timely and improved ordering of drugs and supplies, sensitization to and improved counselling of clients and better communication with district managers to improve implementation. In Tanzania, QI teams also started to access funds collected as part of the national cost-sharing strategy and from community health funds, which had been accumulating funds without being used. District QI teams worked on improving (1) the human resource situation, such as requesting new staff or staff to be transferred from the central level, (2) the drug supply from the Medical Stores Department to facilities and (3) the supervision of district managers of primary care facilities and communities to improve quality of care. The change ideas were implemented and tested during a 1–2-month period using locally generated data (Additional file 1: webannex 7 run charts) and widely implemented if internal monitoring suggested improvements.

**Table 2:** Improvement topics

	Tanzania			Uganda		
	Facility	Community	District	Facility	Community	District
Primary outcomes						
Facility delivery Indicator: women reporting having delivered their last baby in a facility	-Promotion of birth preparedness at ANC -Tracking of home birth together with community volunteers -Male involvement/encourage men to come for ANC for joint counselling (November 2011/February 2012–April 2014^*^)	-Encouragement of facility delivery through home visits and community meetings -Engagement with traditional birth attendants -Escort women to facility in labour -Community fines to family who delivered at home (November 2011/February 2012–April 2014)	-Health managers emphasised importance of health facility delivery during contacts with facility staff	-Encouragement of facility delivery and birth preparedness during ANC counselling (November 2011/January 2013–April 2014)	-Community sensitisation on the importance of birth preparedness and facility delivery during village meetings and religious gatherings -Birth preparedness checklist for each pregnant woman used during home visits -Home visits to ensure items are bought (at least 3 visits) Male involvement—sensitization to bring men to ANC -Follow up pregnant mother using EDD -Referral of pregnant mother in labour to nearby facility with referral note (November 2011/February 13–April 2014)	-Facility delivery improvement discussed during the quarterly district meetings (November 2011–April 2014)
Immediate Breastfeeding Indicator: women reporting breastfeeding within 1 h	No action	No action	No action	-Mother and baby kept together after delivery -Mother shown how to attach and breastfeed by health worker (January 2013–April 2014)	No action	No action
Uterotonic within 1 min after birth Indicator: population indicator “effective coverage” of uterotonics within 1 min after birth	-Orientation of staff Pre-loading of syringe -Improved ordering (November 2011/February 2012–April 2014)	No action	-Support to improved ordering of oxytocin Revision of ordering document and flow -Purchase of oxytocin using district resources (basket funds) (February 2012–April 2014)	-Orientation (on-job training) of health workers on AMSTL -Continuous education sessions on AMSTL Creation of a column in the delivery register for recording AMSTL -Preparation and ensuring that oxytocin is kept/replaced in labour suite each morning -Health Centre IIs request for oxytocin from Health centre IVs (January 2012/January 2013–Apr 2014)	No action	-Ensure adequate supplies of oxytocin in Health Centres III, IV and in a hospital -Permission for Health Centre IIs to request for oxytocin from Health Centre IIIs and IVs -Work with Health Centre IIIs and IVs to include oxytocin forecasts for health centre IIs in their requisition (January 2012–April 2014)
Knowledge of pregnancy danger signs Indicator: mothers reports of knowledge	Danger signs counselling during ANC (February 2013–April 2014)	-Home visits by volunteers (February 2013–April 2014)	District managers mentored and supported health staff (February 2013–April 2014)	Counselling on danger signs during ANC (February 2012/February 13–April 2014)	Counselling on danger signs during home visits (February 2012/July 13–April 2014)	No change idea Community knowledge on danger signs presented on a quarterly basis to district team through report cards
Knowledge of newborn danger signs Indicator: mothers reports of knowledge		Home visits by volunteers (July 2012–April 2014)	District managers mentored and supported health staff (July 2012–April 2014)	Counselling on danger signs during ANC (February 2012/February 2013–April 2014)	Counselling on danger signs during home visits (February 2012/February 2013–April 2014)	No change idea Community knowledge on danger signs presented on a quarterly basis to a district team through report cards
Other/improvement topics chosen by teams						
Post-partum care indicator: mothers’ reports on timing of post-partum care	Keeping women 48 h post-partum in facilities -Counselling of importance of PNC during ANC (November 2011/February 2012–April 2014)	-Home-based counselling on importance of early post-partum care and getting births and birth certificates -Counselling of men to prepare for PNC (November 2011/February 2012–April 2014)	District managers mentored and supported health staff (February 2012–April 2014)	-Give mothers appointment dates for PNC before discharge -Immunise baby but retain Child Health Card till mother comes back for PNC -Health education of mothers on importance of PNC -Re-design clinic so that PNC is held on separate days from ANC -Young child clinic days -Work with VHTs to remind mothers to go for PNC at health facility (November 2011/January 2013–April 2014)	Follow up of all pregnant women (home to home visits) for PNC Community sensitisation on PNC in places of worship Use village leaders to enforce PNC attendance (November 2011/July 2013–April 2014)	No change idea PNC coverage reported on a quarterly basis to district team through report cards
Clean birth kits Indicator: women report having prepared clear birth kits in home deliveries	Educating women of what to purchase for a safe delivery during ANC	Home-based counselling on birth preparedness (April 2012–April 2014)	Support and mentoring	Merged with birth preparedness counselling during ANC	Counselling on birth preparedness during home visits (April 2012/January 2013–April 2014)	
Helping babies breathe, drying and wrapping of babies Indicator: mothers report immediate wrapping of babies during last birth	Helping Babies Breathe training (March 2013–April 2014)	No action	Support and mentoring	No action	No action	No action
ANC 4+ Indicator: mothers report four ANC visits	No action	No action	No action	Sensitisation on importance of four ANC visits Work together with CHWs to promote 4 ANC visits (November 2011/January 2013–April 2014)	Community sensitization through village meetings, religious gatherings Sensitise men to support their spouses to go for ANC (male involvement) Sensitise women on importance of ANC during home visits CHWs refer mother to HF for ANC (with referral note) (November 2011/July 2013–April 2014)	No action
BCG immunisation Indicator: mothers reports of	No action	No action	No action	On-job training and CME for staff on vaccination Do not discharge mother till baby is vaccinated (April 2012/January 2013–April 2014)	Follow up all deliveries in the community to ensure that babies are immunised Home visits to verify immunisation through child cards Community sensitisation meetings on importance of immunisation (April 2012/February 2013–April 2014)	-Support for projection, requisition and procurement of vaccines -Repair all spoilt fridges in health facilities -Ensure that all facilities have gas for the fridges (April 2012/February 2013–April 2014)
Infection prevention Indicator: infection prevention items included clean running water, disinfectant, soap and gloves available in delivery room	Re-organisation of labour room, ordering of supplies using locally available funds (July 2013–April 2014) Reorientation of staff on prophylactic antibiotics for Caesarean section in the district hospital (Nov 2011–April 2014)	Recognition of danger signs for infection together with danger signs post-partum (July/August 2013–April 2014)	Support to mobilising local funds for purchasing supplies for infection prevention (July 2013–April 2014)	No action	No action	No action
Supervision Indicator: documentation of supervision visit from district managers in past 6 months	No action	No action	-Improved roster management -Earmarking of district funds for fuel -Supported facility staff to improve documentation of supervision visits including dates visited, purpose and cadres of the supervisors (November 2013–April 2014)	No action	Community QI teams to be supervised by Health Assistants on a monthly basis (November 2011/February 2013–April 2014)	District QIT to support facility QITs on monthly basis during mentoring and on quarterly basis during learning sessions (November 2011–April 2014)
Other improvement topics						
Syphilis testing	Improved ordering of testing materials (Nov 2011–March 2012 in pilot division)					
Timely and correct use of partographs	-On-job training of staff on partograph use -Copying of partograph forms (August 2013–April 2014)	No action	No action	-On-job training of staff on partograph use -Request for partographs from District Health office -Reminder on labour ward wall on use of partograph -Photocopy partographs for use Keep partographs in labour suite for easy access (November 2011/January 2013–April 2014)	No action	Support requisition and procurement of partographs Photocopy partographs to be supplied to facilities (November 2011–April 2014)

*ANC* Antenatal care, *EDD* Expected date of delivery, *AMSTL* Active management of the third stage of labour, PNC Postnatal care, *CHWs* Community health workersHFs Health facilities, *BCG* Bacillus Calmette–Guérin, *CME* Continuous medical education, *QIT* Quality improvement team,*QITs* Quality improvement teams

*the scale-up of the topic took place between November 2011 and February 2012, the intervention was implemented up to April 2014

In intervention districts, two external medical and social science experts were employed to facilitate learning sessions and mentoring and coaching. Additionally, in each district, three staff members from the health and community sectors, and a varying number of government-salaried staff working at the lower level of the district, were involved in mentoring and coaching and were remunerated through daily allowances as per government guidelines. In Tanzania, these mentors supported one district and 32 health facility QI teams, as well as about 300 village volunteers organised in 10 cluster QI teams. In Uganda, the mentors supported one district, 30 health facility and 61 parish QI teams (see annex II of [23]).

### Outcomes

Our primary coverage outcomes were (1) the percentage of women delivering in a health facility and (2) breastfeeding within 1 h after delivery, as assessed through the continuous household survey using reports from women of reproductive age with a live birth in the 12 months before the survey (Table 3).

**Table 3:** Effects of EQUIP on coverage, quality and knowledge of danger signs

	Tanzania					Uganda			
	*N* (women of reproductive age, 6 rounds)	Survey rounds^a^	1st and 6th round estimates % (95% CI)		Estimated difference-in-difference (95% CI)	*N* (6 rounds)	1st and 6th round estimates % (95% CI)		Estimated difference-in-difference (95% CI)
			Intervention	Comparison			Intervention	Comparison	
Primary coverage indicators									
Facility delivery	1422	1st round 6th round	55 (45 to 65) 87 (77 to 93)	62 (50 to 72) 78 (67 to 86)	7 (−7 to 21)	2929	56 (47 to 64) 68 (58 to 76)	31 (25 to 39) 42 (33 to 51)	−3 (−15 to 9)
Immediate breastfeeding	1398	1st round 6th round	31 (22 to 42) 37 (28 to 47)	32 (21 to 46) 40 (30 to 52)	−7 (−21 to 7)	2793	37 (30 to 45) 41 (35 to 46)	20 (16 to 26) 23 (18 to 29)	−6 (−17 to 5)
Primary quality indicator ^b^									
Population indicator “effective coverage” of uterotonics within 1 min after birth	409 last events 1422 live births^b^	1st round 6th round	29 (16 to 41) 81 (72 to 91)	44 (31 to 58) 70 (58 to 81)	26 (25 to 28)	291 last events 2929 live births^b^	38 (27 to 50) 59 (48 to 70)	11 (3 to 20) 23 (10 to 6)	8 (6 to 9)
Primary knowledge indicator (assessed through interviews with women of reproductive age with a live birth in the year prior to the survey)									
Mothers’ knowledge of critical danger signs pregnancy^c^	1422	1st round 6th round	25 (18 to 33) 45 (36 to 54)	40 (30 to 51) 45 (34 to 56)	4 (−11 to 18)	2993	36 (30 to 42) 49 (43 to 55)	32 (27 to 39) 43 (35 to 40)	−2 (−14 to 11)
Mothers’ knowledge of critical danger signs newborns^c^	1422	1st round 6th round	36 (29 to 45) 38 (30 to 48)	35 (26 to 45) 34 (26 to 43)	2 (−12 to 15)	2848	45 (40 to 50)~ 34 (28 to 40)	38 (33 to 43) 27 (21 to 34)	−7 (21 to 6)
Secondary coverage indicators (indicators monitoring improvement topics chosen by the teams)									
Post-partum care <7 days (restricted to home births)	442	1st round 6th round	19 (11 to 30) 23 (10 to 46)	27 (14 to 47) 23 (7 to 54)	17 (−8 to 40)	1103	4 (1 to 12) 3 (1 to 10)	3 (1 to 7) 2 (1 to 8)	−3 (−8 to 2)
Clean birth kit (restricted to home births)	442	1st round 6th round	15 (7 to 29) 62 (23 – 84)	23 (13 to 37) 23 (11 to 41)	31 (2 to 60)	1103	9 (3 to 22) 25 (18 to 22)	5 (2 to 9) 7 (3 to 15)	10 (−6 to 26)
Wrapping of babies (as part of HBB)	1288	1st round 6th round	43 (33 to 53) 56 (48 to 65)	44 (34 to 56) 33 (25 to 44)	7 (−21 to 36)	Not prioritised by QI team			
ANC 4 +		1st round 6th round	Not prioritised by QI team			2990	41 (35 to 48) 47 (40 to 54)	34 (28 to 39) 38 (31 to 46)	0 (−15 to 15)
BCG immunisation of newborns		1st round 6th round				1654	81 (73 to 88) 81 (73 to 88)	77 (71 to 83) 84 (78 to 88)	−8 (−16 to 0)
Secondary quality indicators (indicators monitoring improvement topics chosen by the teams) (assessed through health facility assessments)									
Infection prevention items available^d^	352	Baseline Endline	13 (4 to 34) 69 (50 to 83)	48 (27 to 67) 76 (58 to 87)	21 (−4 to 46)				
Supervision to health facilities (past 6 months)	354	Baseline Endline	78 (57 to 91) 100	92 (73 to 98) 100	14 (0 to 28)				

^a^First round November 2011 to February 2012 included 111 and 91 women with a live birth in the year prior to the survey in intervention and comparison districts in Tanzania and 238 and 272 in Uganda, respectively. The sixth round January 14–April 14; ~relates to the second round April 2012 to July 2012 included 106 and 101 women with a live birth in the year prior to the survey in intervention and comparison districts in Tanzania and 281 and 199 in Uganda, respectively.

^b^Assessed through multiplying household survey coverage estimates of facility delivery in women with a live birth in the last year prior to the survey to reports of health workers on implementation practices in surveyed facilities using the *last event* module during the same time period. We included 409 last event questionnaire in Tanzania and 291 in Uganda.

^c^Knowledge of all three critical danger signs in pregnancy (severe vaginal bleeding, oedema of face/hands and blurred vision) and four in newborns (convulsions, difficult breathing, lethargy/unconsciousness and very small baby)

^d^Infection prevention items included clean running water, disinfectant, soap and gloves

The primary quality outcome indicator was the proportion of births in which an uterotonic drug was administered within 1 min after delivery. This indicator was constructed by multiplying women’s reports of the place of birth assessed through household surveys with reported health workers practicing on the usage of uterotonics in surveyed facilities. The health worker’s report was based on a *last event* module that asked staff providers about their practices in a narrative and non-threatening way [30]. As this measurement mode is not validated in low-resource settings, we did observations of delivery practices in selected facilities to validate health worker reports of implementation practices [34].

The primary knowledge indicator was the proportion of women who knew danger signs both in pregnancy and for newborns, as measured amongst mothers who gave birth in the year before the continuous household survey. We used open-ended questions to assess knowledge of danger signs, which was defined as recalling all three critical signs of severe vaginal bleeding, oedema of the face/hands and blurred vision/headache in pregnancy and all four critical signs in newborns (convulsions, difficult breathing, lethargy/unconsciousness and very small baby) [23]. The secondary outcomes were seven indicators that were constructed to reflect the improvement work such as post-partum care and clean birth kits (Tables 2 and 3).

### Sample size

Assuming a design effect of 1.4 and 10% refusals, the size of the survey was calculated to provide at least 80% power to detect small absolute increases (fewer than 10 percentage points) between the beginning and end of the intervention period for outcomes across the continuum of care, 10-percentage-point increases each year of the intervention for most outcomes (including institutional delivery and immediate breastfeeding) and larger increases more frequently. See Marchant et al. [30] for a detailed discussion.

### Survey data collection

We implemented continuous household cluster and health facility surveys in the intervention and comparison districts. The details are presented elsewhere [30]. Briefly, questionnaires were developed based on well-established sequences of questions as used in Demographic and Health Surveys and Service Provision Assessments [35] and also drew upon earlier work in the study areas [5, 36]. Data collection was organised in 5-month cycles, comprising a 4-month ‘round’ of field work and a 1-month break for aggregated analysis and planning for the next cycle.

Based on a sampling frame of the lists of sub-villages with the total number of households (Tanzania) and parish-level lists (Uganda), we sampled, each month, and in each district, 10 clusters (comprised of 300 households) using probability proportional-to-population-size sampling. We thus included no repeated sample of the same women over time but 24 independent probability samples of household clusters to represent each district each month while. We systematically selected each cluster of 30 households from a household list using a fixed fraction (total number of households in the sub-village divided by 30) [30]. After completion of 4 months of data collection, data were aggregated (1200 households and an estimated 152 women with a recent birth, per district) for analysis, and report cards were prepared (Additional file 1: webannex 5 report card). Each 4-month round also included a census of all health facilities to assess readiness and also included a *last event* module whereby the birth attendant for the last birth recorded in the health facility register was identified and interviewed about the care they had given during that birth.

### Context analysis

The contextual factors were assessed using pre-defined indicators based on the health system building block framework to inform the plausibility analysis. The selection of indicators was informed by the work of Victora et al. [31]. Systematic investigation into concurrent context changes is needed to draw any conclusion when randomisation is not feasible [29, 31]. The detailed methodology of our context analysis is described elsewhere [23, 33]. In brief, we included indicators of financing, leadership, human resources, drugs and supplies, health information and service delivery [37]. Data were collected using (1) district reports, (2) interviews every fourth month with the district health managers and (3) the continuous household and health facility surveys. A structured questionnaire addressed to the district medical officer and his or her team collected information on the district planning process including participation of civil society, implementation of district plans and other aspects of leadership and governance [23]. Qualitative information on leadership and governance were triangulated with information available in district reports. Analysis used a qualitative and explanatory approach.

### Survey data analysis

We used an approach adapted from the analytic methods to model data points over time (see Additional file 1: webannex 8 statistical methods). For each of the six time points of the continuous survey rounds, we calculated the difference in the indicator estimate between the intervention and comparison district [38]. We used meta-regression to fit a regression line through the resulting six data points over the 30 months of data collection and used this regression to estimate the difference-of-difference value between intervention and comparison districts from the baseline (first data collection round) to the end line (last data collection round). Estimates were adjusted for the sampling method (using svy commands in STATA). The delta method was used to estimate the variance of the difference-of-differences measure and present confidence intervals [39]. Rather than using a time-series approach based on autoregressive integrated moving average (ARIMA) models, we chose a simpler and more transparent analytical method that we thought was more appropriate for our small number of data points over time. We did, however, perform a sensitivity analysis with ARIMA models to investigate correlation over time and found no significant impact on the results for our primary outcomes. We did not adjust the models for potential confounding factors. We used information on potential confounders qualitatively [29]. Descriptive tabulation was done for the outcomes selected to present the context. Analysis was done using STATA 13 (StataCorp, Texas, USA).

### Ethics

Ethical clearance was obtained from the institutional review boards of Ifakara Health Institute, the Tanzania Commission for Science and Technology, the Uganda National Council of Science and Technology, Makerere University School of Public Health and the London School of Hygiene and Tropical Medicine (LSHTM). This activity underwent human subjects review process at CDC and was approved as not being engaged in human subjects research. Advocacy and sensitization meetings with district and sub-district authorities were held at the start of the project. Communities and health facilities were informed about the survey by a survey team member 1 day prior to the interview, using information sheets in the local languages. Written informed consent to participate in the surveys was obtained from household heads, women, facility in-charge and health staff interviewed.

## Results

Both intervention districts with their health facilities and communities participated in the implementation. Of the eight learning sessions planned during the 24 implementation months, seven and five health facility sessions and six and five community sessions were held in Tanzania and Uganda, respectively. Approximately, 60–75% of the planned monthly mentoring and coaching sessions were implemented (13 and 18 of the 24 planned health facility and 18 and 18 of the 24 planned community visits in Tanzania and Uganda, respectively (Additional file 1: webannex 2).

All of the district, facility and community teams engaged in QI. A staggered implementation was done, starting in one division (an administrative unit below the district level) in Tanzania and one sub-district in Uganda before reaching the remainder of each district (Additional file 1: webannex 3). The teams in Tanzania worked with three of the four improvement topics measured by the primary outcomes while the teams in Uganda worked with all four (Table 2). Full implementation took longer than planned, reaching all sub-districts in March 2012 in Tanzania and January 2013 in Uganda. QI teams in both countries prioritised additional improvement topics, including early postnatal care and preparation of clean birth kits.

### Background characteristics

Of the 14,400 sampled households in each country over the six survey rounds, 14,255 (99%) and 13,125 (93%) households in Tanzania and Uganda, respectively, consented to be included in the survey (Fig. 1). The household interviews listed 13,239 and 14,718 eligible resident women of reproductive age. In Tanzania, 11,835 (89%) women were interviewed and 1415 had a live birth in the 12 months before the survey. In Uganda, 12,870 (87%) were interviewed and 2993 had a live birth in the previous 12 months. Of the 378 and 360 planned facility assessments during the six rounds, 354 (94%) and 302 (84%) were completed in Tanzania and Uganda, respectively. A total of 409 and 291 *last event* interviews were done with health workers in Tanzania and Uganda.

### Effects on demand for and supply of maternal and newborn services

The internal monitoring data shown on the run charts of the QI teams suggested improvements in facility delivery (Additional file 1: webannex 6a and 6b). The combined run charts of the 34 included facilities in Tanzania suggested an increase in the coverage levels of facility births from around 85 to 95% over the implementation period, a 10-percentage-point increase. However, results warrant careful interpretation because home births were not reliably documented in facility records.

**Fig. 2:**
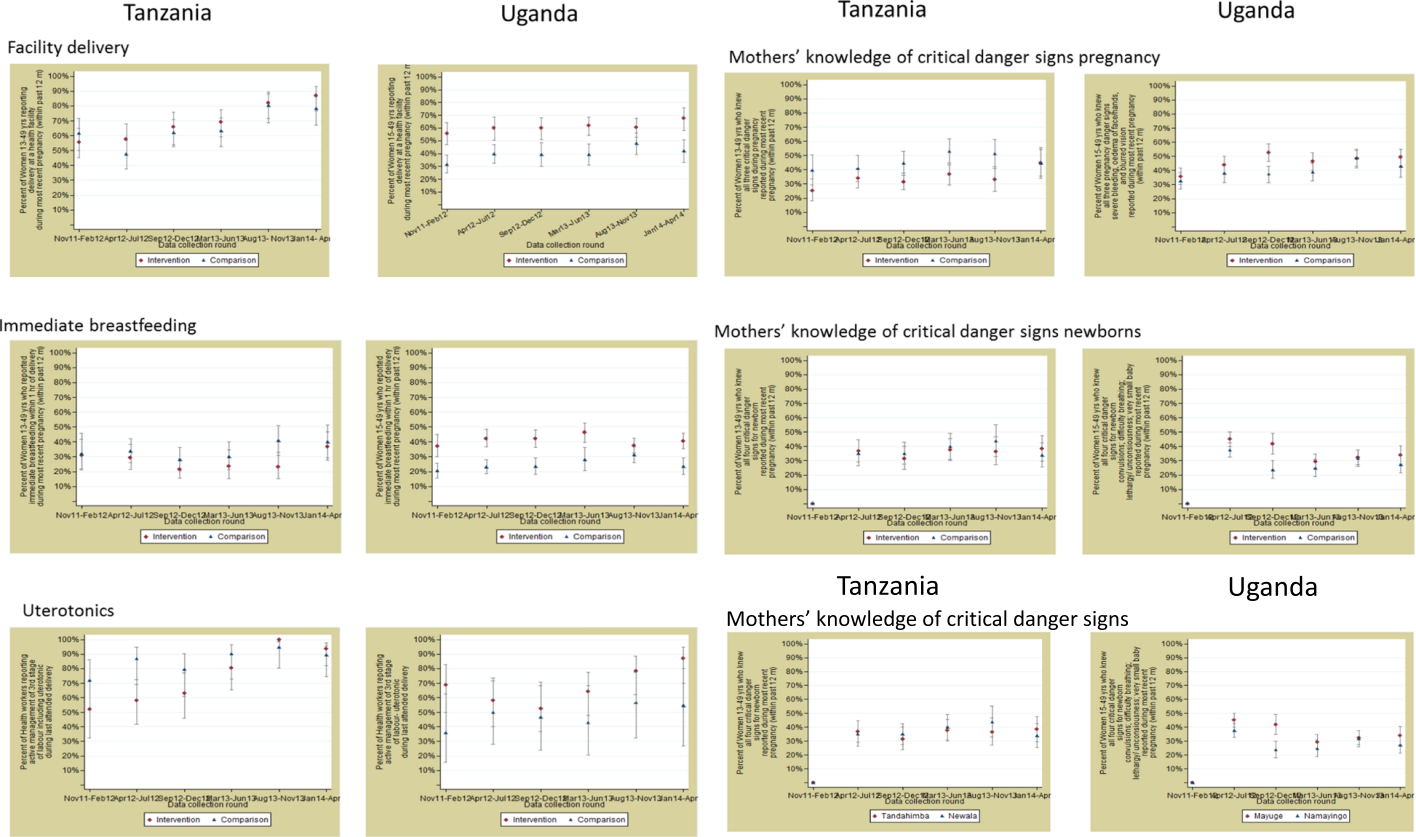
Effects of EQUIP on coverage, quality and knowledge of danger signs

According to the household survey data, facility delivery increased from 55 to 87% in the intervention district and 62 to 78% in the comparison district, suggesting no evidence of any association between the intervention and facility delivery in Tanzania (difference-in-difference 7%; 95% CI −7 to 21%, Table 3, Fig. 2). In Uganda, facility delivery increased from 56 to 68% in the intervention and 31 to 42% in the comparison area, giving no evidence of an association between the intervention and facility delivery (difference-in-difference −3%; 95% CI −15 to 9%).

We found some evidence that the EQUIP approach increased the proportion of mothers who reportedly received uterotonics within 1 min of childbirth. In Tanzania, we observed an increase in the proportion of women with a live birth in the year prior to the survey who received uterotonics, with a difference-in-differences of 26 percentage points (95% CI 25–28%). In Uganda, the difference was smaller at 8 percentage points (95% CI 6–9%). We found no evidence of an association between the EQUIP intervention and the primary outcomes of immediate breastfeeding or knowledge of maternal and newborn danger signs.

In Tanzania, the analysis of the secondary coverage outcomes, chosen to reflect the prioritised improvement topics, indicated some evidence of an association between the EQUIP intervention and birth preparedness through preparation of clean birth kits for home deliveries (31%; 95% CI 2–60%). No evidence of an association was observed for early postnatal care for home deliveries (17%; 95% CI −8 to 40%). There was no evidence of an effect of the intervention on increased availability of selected items for infection prevention in health facilities (21%; 95% CI −4 to 46%). We observed some association between the EQUIP approach and improvements in supervision visits by district managers to primary health facilities (14%; 95% CI 0–28%). In Uganda, we saw no evidence of effect of the intervention on any secondary coverage outcomes.

### Context analysis

The context analysis indicated differences in key health system indicators between intervention and comparison districts and between both countries (Table 1). District reports on finances indicated that in 2013 and 2014, the Tanzanian comparison district had 12 USD per capita for health expenditure, compared to 7 USD in the intervention district, Tandahimba. For Uganda, comparable data were unavailable, but other studies suggest levels around 6 USD per capita for health [40]. In Tanzania, the situation on human resources was better in the intervention district compared to the comparison district (0.97 compared to 0.79 nurses per 1000 population). In Uganda, more human resources were available in the intervention district (0.70 compared to 0.53 nurses per 1000 population). There were few in-service-training sessions in maternal and newborn health in both districts in Tanzania other than those supported by EQUIP. In Uganda, a few training sessions on maternal and newborn health care were provided in the intervention area, supported by other international partners. Information from the comparison area was not available. Availability of drugs and supplies was better overall in Tanzania than those in Uganda. For example, magnesium sulphate was available in the intervention district in Tanzania in the first and sixth round of facility assessments in 21 and 30% of facilities, whereas the respective figures were 3 and 0% in the intervention district in Uganda.

In both countries, health planning was based on health information from the health management information system (HMIS). In Tanzania, the EQUIP data were not included in the formal planning process, as planning guidelines recommend the use of HMIS data alone. In Uganda, however, the district team started to use the EQUIP data in planning in the second year of EQUIP support.

## Discussion

We implemented a comprehensive QI strategy at full implementation for a period of 26 months in Tanzania and 15 months in Uganda, reaching all facilities and communities and cutting across all levels of the district health care system in two rural districts in Tanzania and Uganda. To our knowledge, this is the first evaluation of QI implemented simultaneously at all three levels of a district health system (district managers, health facilities and community level) in a low-income setting. In both countries, we observed an association between the EQUIP approach and only one of the four main outcomes, the proportion of mothers reportedly receiving uterotonics within 1 min after birth. In Tanzania, we also observed an association between the QI strategy and the preparation of birth kits and supervision of health facilities by district managers.

In both countries, the impact of the EQUIP intervention on the implementation of uterotonics immediately after birth was a result of the changes within the facilities (job aids, improved ordering) and strong support from the district health managers (improved drug management), supported by some increase in facility delivery. This suggests that a systemic approach to QI, concurrently addressing bottlenecks in uptake of care, availability of drugs and health worker practice might yield better results. QI initiatives elsewhere have also successfully prioritised uterotonics [15, 41]. Despite the drug supply being a constant concern in the study setting, we nevertheless found evidence of a positive change at a population level.

Work on other supply side outcomes was less successful, possibly because they required changes that were outside the district capabilities. Syphilis screening in antenatal care was an improvement topic in the early phase of implementation in Tanzania. However, the team abandoned this topic because local changes could not overcome the lack of tests at the local medical stores department caused by national level shortages. This was in contrast with supply constraints for oxytocin. Oxytocin was available in sufficient quantities at the local medical stores, and the supply chain bottleneck was solely at district level. The increase in availability at facilities of oxytocin compared to syphilis tests as documented by our context analysis supports this interpretation. This example illustrates both the opportunity and limitation of QI at the district level and supports the need to combine district improvement work with national health system strengthening.

Improvements were not observed for other primary outcomes; we found no evidence of an effect of the EQUIP intervention on knowledge of danger signs and immediate breastfeeding amongst mothers with a recent birth. While immediate breastfeeding was a pre-defined indicator, it was not prioritised by the health facility teams in Tanzania as they unanimously perceived that health workers already facilitated immediate breastfeeding in facilities and further emphasis was not needed, although mothers’ reports suggested low implementation levels. The standard evaluation approach of reporting results for pre-defined outcomes was therefore not entirely congruent with the evolving nature and bottom-up approach of QI. The EQUIP intervention had a partly self-directed nature and left us with the dilemma of reporting according to a pre-defined plan, as faced by others [42–44].

Our QI approach differed from other QI interventions in relation to the operationalisation and priority topics as well as in relation to evaluation methodology. Most improvement initiatives are restricted to facilities and include highly selected improvement topics, which was not the case in our study [15, 18]. A few studies report QI strategies with elements similar to ours (targeting maternal and newborn services and including community work) [19, 21], but differences in the overall strategy, the evaluation design and methodology of analysis precludes meaningful comparisons of results.

Both population-based and facility data indicated secular improvements in intervention and comparison areas such as for facility delivery in both countries and availability of drugs and supplies in Tanzania. For some indicators, we provided evidence of a positive improvement greater than the secular change in the intervention area. Without the data from our comparison district, we might have misinterpreted secular changes as improvements due to our strategy.

We found relatively little evidence of the effect of the intervention on several outcomes. In Tanzania, we found no evidence of an association between the EQUIP intervention and either facility delivery or post-partum care, both of which were prioritised by the QI teams. This may be due to a lack of power to detect changes of less than 10 percentage points. QI might also be more adequate to improve the supply side than demand side.

We saw greater effect in Tanzania than in Uganda. A team of similar size and with similar resources to that in Tanzania had to reach two-and-a-half times the number of pregnant women and their newborns. It is therefore likely that any intervention effect in the facilities and communities in Uganda was more diluted than in Tanzania. However, there were also contextual factors which might explain differences between Tanzania and Uganda [45–47]. District resources were more limited in Uganda than those in Tanzania where pooled basket funding of approximately one USD per capita is made available to districts and can be spent on local priorities [40]. Improvement teams in Tanzania also tapped into other local resources available through the scale-up of community health funds and other insurance schemes [48], and this might have led to greater improvements in the availability of oxytocin, infection prevention items and supervision in Tanzania compared to those in Uganda. As drugs and supplies are crucial not just to provide quality care, but also to keep health workers motivated and increase community demand, this could be an additional factor. Although QI can overcome low implementation levels to some extent by optimising the use of available resources, our study suggests a limitation of QI, in that a certain amount of autonomy, locally available funds and a reasonably functioning drug-distribution system is also likely to be needed for QI strategies to reach their full potential.

### Strengths and limitations

Our study has four major strengths. Firstly, it is to our knowledge the first to conduct QI at all levels of a district health system, including district management, all primary and referral facilities and communities through a network of volunteers and community health workers. Secondly, we used a plausibility design as recommended for health systems interventions [29, 31] which provides more robust evidence than a before-and-after design. Moreover, our context analysis helped to interpret findings from a plausibility perspective [29]. Thirdly, we evaluated the QI intervention using independently generated population- and facility-based data in both intervention and comparison areas. Fourthly, the same intervention was implemented in two different countries, which provided learning in different contexts.

Our study also has limitations. Although the survey team collected data that were independent from the QI teams, they were not masked. However, we think it unlikely that interviews with mothers in intervention and comparison areas or in health facilities differed systematically, because the survey team operated independently and were unaware of the chosen outcome indicators.

We implemented QI in only one intervention district in each of the two countries, and our quasi-experimental design limits both generalisability and internal validity. Our context analysis showed differences between intervention and comparison districts. While these differences did not point to a clear advantage of either district in Tanzania, the implementation district in Uganda seemed to be better equipped in terms of human resources and drugs and supplies, biasing the results in favour of the intervention.

For the analysis over time, our household surveys were powered to detect statistically significant changes of 10 percentage points or greater, which may have contributed to the lack of evidence of associations between the EQUIP approach and improved outcomes. Also, we had a limited number of data points to be included in the analysis over time.

The methods we used to assess changes in knowledge of danger sign and breastfeeding within 1 h are subject to measurement biases, despite our use of a well-established sequence of questions [49]. Misinterpretation of breastfeeding questions, for example, has been reported [50]. For many important intrapartum quality indicators, such as uterotonics after childbirth, no standard method of measurement is available. We used *last event* reports of health workers; however, their reliability can be questioned. However, a small add-on observation study in the implementation district confirmed implementation levels reported by health workers. Moreover, we considered injection of an uterotonic within 1 min as the most important aspect of Active Management of the Third Stage of Labor as supported by the literature [51] and omitted two other aspects of controlled cord traction and uterine massage.

We phased short implementation period, coupled with the prolonged roll-out in a larger implementation district in Uganda than Tanzania. The issues of relatively low implementation strength and short duration were exacerbated by the use of women’s report to establish coverage. In household surveys, mothers are commonly asked to report on births that occurred within a defined period before the interview date, thus providing information about the past. We used a recall period of 12 months prior to the date of assessment for population indicators, which meant that only the last survey round covered a time period where all teams were active, but not all improvement topics were implemented for this time period (Additional file 1: webannex 3).

This design constraint might have biased out the results versus the nullhyposthesis.

For QI to have an impact, sufficient input and support is required. Many of the change ideas were only fully implemented after a 3-month piloting phase so that implementation in all facilities and communities was achieved only 6–12 months before the end of the project. Improvement ideas need to mature, and it often takes several months for an improvement topic to reach levels above 90% [15]. Moreover, our intervention might have had too little focus on the improvement areas for which we had pre-defined primary indicators as we allowed the districts to prioritise improvement topics from a much broader pre-defined list shown in the improvement charter. Finally, there was some misalignment of QI topics teams worked on and primary outcomes for breastfeeding in Tanzania.

## Conclusions

We implemented a comprehensive QI intervention addressing all levels of a district health system to improve implementation of essential interventions for maternal and newborn care in Tanzania and Uganda. We found an association between our QI approach and improved implementation levels for only one of our four main outcomes (women receiving oxytocin within 1 min after birth) in both countries. In addition, statistically significant associations were seen for the outcomes of preparation of birth kits and supervision of health facilities by district managers in Tanzania. However, we found no evidence of population-level impact on other outcomes. Reasons for the lack of effects included limited implementation strength as well a relatively short follow-up period in combination with a 1 year recall period for population-based estimates and a limited power of the study to detect changes smaller than 10 percentage point.

## Additional file

Additional file 1:
**Webannex I** EQUIP Maps. **Webannex II** EQUIP mentoring and coaching. **Webannex III** EQUIP Timeline of assessment and implementation. **Webannex IV** Project charter. **Webannex V** EQUIP Example report card. **Webannex VI** Vignettes. **Webannex VII** EQUIP Example Runchart. **Webannex VIII** EQUIP Example Analysis. (ZIP 1064.96 kb)

## Abbreviations

CI: Confidence interval
EQUIP: Expanded quality management using information power
HMIS: Health management information system
PDSA: Plan-do-study-act
QI: Quality improvement
USD: United States dollars
