# Supplementary material for: Effects of the EQUIP quasi-experimental study testing a collaborative quality improvement approach for maternal and newborn health care in Tanzania and Uganda
Source: Implement Sci. 2017 Jul 18;12:89. doi: 10.1186/s13012-017-0604-x (PMC5516352; doi:10.1186/s13012-017-0604-x)
Supplement: Additional file 1: — Webannex I EQUIP Maps. Webannex II EQUIP mentoring and coaching. Webannex III EQUIP Timeline of assessment and implementation. Webannex IV Project charter. Webannex V EQUIP Example report card. Webannex VI Vignettes. Webannex VII EQUIP Example Runchart. Webannex VIII EQUIP Example Analysis. (ZIP 1064.96 kb) [file 13012_2017_604_MOESM1_ESM.zip › Webannex II EQUIP mentoring and coaching.docx]

**Webannex 2: Table Mentoring and coaching, learning sessions, and District QI team meeting frequencies per quarter**

| No. | ACTIVITY | FREQUENCY (Quarter of project implementation) | | | | | | | |
| --- | --- | --- | --- | --- | --- | --- | --- | --- | --- |
|  | Activity | Q1  May’12- Jul’12 | Q2  Aug’12-Oct’12 | Q3  Nov’12-Jan’13 | Q4  Feb’13-Apr’13 | Q5  May’13-Jul’13 | Q6  Aug’13-Oct’13 | Q7  Nov’13-Jan’14 | Q8  Feb’14-April’14 |
| TANZANIA | | | | | | | | | |
| **1.** | Health Facility mentoring and coaching | 1 | 0 | 2 | 2 | 2 | 2 | 2 | 2 |
| **2.** | Community mentoring and coaching sessions | 1 | 2 | 3 | 1 | 2 | 2 | 2 | 3 |
| **3.** | Health facility learning sessions | 1 | 0 | 2 | 1 | 1 | 1 | 0 | 1 |
| **4.** | Community learning sessions | 1 | 1 | 0 | 1 | 1 | 1 | 0 | 1 |
| **5.** | District QIT meetings | 1 | 2 | 1 | 1 | 1 | 2 | 2 | 1 |
| UGANDA | | | | | | | | | |
| **1.** | Health Facility mentoring and coaching | 2 | 3 | 1 | 2 | 3 | 2 | 3 | 2 |
| **2.** | Community mentoring and coaching sessions | 2 | 3 | 1 | 2 | 3 | 2 | 3 | 2 |
| **3.** | Health facility learning sessions | 1 | 0 | 1 | 1 | 0 | 1 | 0 | 1 |
| **4.** | Community learning sessions | 0 | 1 | 1 | 1 | 0 | 1 | 0 | 1 |
| **5** | District QIT meetings | 1 | 3 | 0 | 1 | 2 | 1 | 3 | 1 |
