# Supplementary material for: Effects of the EQUIP quasi-experimental study testing a collaborative quality improvement approach for maternal and newborn health care in Tanzania and Uganda
Source: Implement Sci. 2017 Jul 18;12:89. doi: 10.1186/s13012-017-0604-x (PMC5516352; doi:10.1186/s13012-017-0604-x)
Supplement: Additional file 1: — Webannex I EQUIP Maps. Webannex II EQUIP mentoring and coaching. Webannex III EQUIP Timeline of assessment and implementation. Webannex IV Project charter. Webannex V EQUIP Example report card. Webannex VI Vignettes. Webannex VII EQUIP Example Runchart. Webannex VIII EQUIP Example Analysis. (ZIP 1064.96 kb) [file 13012_2017_604_MOESM1_ESM.zip › Webannex IV Project charter.docx]

**Webannex 4: Project Charter**

Improving Utilization and Quality of Care to Reduce Maternal and Neonatal Mortality in Tandahimba District, Southern Tanzania and Mayuge District, Uganda

Project Charter

***Background***

Maternal and Newborn health is still a major problem in Tanzania and Uganda; both countries have developed a plan setting out how to address these challenges.

In Tanzania, the National Road Map Strategic Plan (2008)^[[1]](#footnote-1)^ aims to address maternal, newborn and child health and accelerate mortality reduction in an integrated manner across the continuum of care. Its vision is that of a healthy and well-informed Tanzanian population who have access to quality, affordable and sustainable maternal, neonatal and child health services. By 2015 the objectives of this plan are to reduce maternal mortality from 578 to 193 per 100,000 live births, and to reduce neonatal mortality from 32 to 19 per 1000 live births.

In Uganda, there is also a focus on accelerating the reduction of maternal and neonatal morbidity and mortality. This is aimed at helping achieve Millennium Development Goals 4 and 5 which are to reduce neonatal mortality rate from 29 in 2006 to <20 deaths per 1000 live births by 2015 and maternal mortality from 435 in 2006 to 130 deaths per 100,000 live births by 2015 respectively^[[2]](#footnote-2)^.

***The Challenge***

Many effective maternal and newborn health interventions are available, but coverage remains limited^[[3]](#footnote-3)^ Both demand- and supply-side factors critically determine coverage and the effectiveness of interventions, and opportunities to prevent and treat poor health are lost at multiple steps. Preventive and curative services do not reach the people in need, providers do not always examine patients properly or follow available standards and guidelines, drugs are out of stock or of poor quality, or users do not comply with recommendations. This results in low effectiveness at patient or community level of potentially highly effective interventions.

***Project Mission***

The Project’s mission is to support the Tanzanian and Ugandan Ministries of Health in the realization of their goals to improve maternal and newborn survival by increasing the quality and utilisation of health care services for mothers and newborns using quality improvement approaches in a scalable and sustainable manner. In engaging with communities, health facilities and district management teams, the project will help to address demand and supply side challenges alongside systems-wide constraints. The basic collaborative design will be enhanced through a complementary strand of activity designed to generate and feed back high quality, locally relevant information through continuous household and health facility surveys.

***Collaborative Goals***

Whilst the overall goal of the of the project is to reduce maternal and neonatal mortality in the intervention districts of Tandahimba and Mayuge, it will not be possible to demonstrate reductions through direct measurement without sample sizes far beyond the scope of this project. Modelling tools (LiST) will therefore be deployed to estimate the overall impact of changes in maternal and neonatal deaths from measured changes in utilization and quality of health services.

***Indicators***

The indicators - as listed in the table below – include indicators measuring progress in relation to the high priority interventions but not ‘lower priority’ interventions. Still countries and quality teams might choose based on better local knowledge intervention rated as lower priority. The list of indicators the team uses will then be expanded. Thus the list should be viewed as an essential indicator list which might be expanded if needed.

The wording of indicators is based on the wording used in the household survey indicator list. Indicators based on the health facility records, will use as denominator ‘expected birth in calendar year or other defined time periods’ rather than a live births in the two years prior the survey. Still the definition of the nominator should be the same or very similar for most health facility record based indicators.

It is important to note that the indicators do not equal the extent of the interventions. The interventions might include more than what is measured by the indicators. For example, birth preparation might include advice on postnatal care, although this is not explicitly included in the definition of the indicator.

| **Utilisation Goal 1** | ***to increase the numbers of women attending four or more ANC consultations in Tandahimba and Mayuge Districts by x% by April 2014*** | | | | | |
| --- | --- | --- | --- | --- | --- | --- |
| **Indicator ^1^** | **Current performance (Uganda)** | | **Current performance (Tanzania)** | | **Goal (Uganda)** | **Goal (Tanzania)** |
| ***ANC coverage*: attendance at least 4 times during pregnancy - the proportion of women with a live birth in the last 24 months who were attended by any health worker for antenatal care at least four times while pregnant** | 47.2% ^[[4]](#footnote-4)^ | | 42.7%^[[5]](#footnote-5)^ | | 70% (2015)^[[6]](#footnote-6)^ | 90% (2015)^[[7]](#footnote-7)^ |
| **Utilisation Goal 2** | ***to increase the numbers of skilled deliveries in Tandahimba and Mayuge Districts by x % by April 2014*** | | | | | |
| **Indicator** | | **Current performance (Uganda)** | **Current performance (Tanzania)** | | **Goal (Uganda)** | **Goal (Tanzania)** |
| Percent of live births in the five years preceding the survey delivered in a health facility  Percent of live births in the five years preceding the survey delivered by skilled provider  ***Institutional delivery:* The proportion of women aged 15‐49 with a birth in the 24 months preceding survey who delivered in a health facility**  ***Skilled attendant at delivery*: The proportion of women aged 15‐49 with a live birth in the 24**  **months preceding survey who were attended during childbirth by a skilled health personnel** | | 54.2% (East Central)^[[8]](#footnote-8)^  55.6 (East Central) ^[[9]](#footnote-9)^ | 58.6% (Mtwara)^[[10]](#footnote-10)^  59.5% (Mtwara)^[[11]](#footnote-11)^  69.3%  67.0% | | 90% (2015)^[[12]](#footnote-12)^ | 80% (2015) ^[[13]](#footnote-13)^ |
| **Utilisation Goal 3** | | ***to increase the caesarean section rate in Tandahimba and Mayuge Districts by x% by April 2014*** | | | | |
| **Indicator** | | **Current performance (Uganda)** | **Current performance (Tanzania)** | | **Goal (Uganda)** | **Goal (Tanzania)** |
| Percent of live births in the five years preceding the survey delivered by caesarean-section  **Proportion of all deliveries that were caesarean sections during the last 4 months** | | 5.8% ^[[14]](#footnote-14)^(Mtwara) | 2.8 (East Central)^[[15]](#footnote-15)^ | | ***TO BE DEFINED LOCALLY*** | ***TO BE DEFINED LOCALLY*** |
| **Utilisation Goal 4** | | ***to increase the numbers of women and newborns who receive post-natal care within 48 hours of delivery by x% by April 2014*** | | | | |
| **Indicator** | | **Current performance (Uganda)** | | **Current performance (Tanzania)** | **Goal (Uganda)** | **Goal (Tanzania)** |
| ***Postnatal check: Number of women and their babies* who receive postnatal care within two days of childbirth** | | 23.8% (Central East)^[[16]](#footnote-16)^ | | 45% ^[[17]](#footnote-17)^(Mtwara) | ***TO BE DEFINED LOCALLY*** | ***TO BE DEFINED LOCALLY*** |

| **Quality Goal 1** | ***x% of pregnant women in their 3rd trimester who report having a birth preparedness plan that includes transport arrangements for an emergency, clean cloth for drying & wrapping baby & clean blade and new thread for umbilical cord*** | | | | |
| --- | --- | --- | --- | --- | --- |
| **Indicator** | **Current performance (Uganda)** | | **Current performance (Tanzania)** | **Goal (Uganda)** | **Goal (Tanzania)** |
| Percentage of mothers who delivered at home made preparation for birth by buying soap  Percentage of mothers who delivered at home prepared a new or washed cloth for drying the child  Percentage of mothers who delivered at home had made plans in case of an emergency during delivery  Percentage of mothers who delivered at home cut the cord with a new razor blade  Percentage of mothers who delivered at home tied the cord with a new thread.  ***Content of antenatal care: birth preparedness*. The proportion of women aged 15-49 who had a live birth in the 24 months preceding survey and who received antenatal care who report that they were advised about birth preparedness during antenatal care** |  | | 84%^[[18]](#footnote-18)^  85%^[[19]](#footnote-19)^  58%^[[20]](#footnote-20)^  91.2%^[[21]](#footnote-21)^  49%^[[22]](#footnote-22)^ |  |  |
| **Quality Goal 2** | **x% of pregnant women are tested at least once for syphilis** | | | | |
| **Indicator (proposed (in bold))** | **Current performance (Uganda)** | **Current performance (Tanzania)** | | **Goal (Uganda)** | **Goal (Tanzania)** |
| **The proportion of women aged 15‐49 who had a live birth in the 24 months preceding survey and who report that they were tested for syphilis at least once during antenatal care** |  |  | |  |  |
| **Quality Goal 3** | **x% of pregnant women able to cite at least two danger signs of pregnancy** | | | | |
| **Indicator** | **Current performance (Uganda)** | | **Current performance (Tanzania)** | **Goal (Uganda)** | **Goal (Tanzania)** |
| Percentage of women who received antenatal care for their most recent birth, who were informed of signs of pregnancy complication  Percentage of population with knowledge of at least 2 danger signs in pregnancy and child birth | 21.3% (East Central)^[[23]](#footnote-23)^  40% (2005)^[[24]](#footnote-24)^ | | 45% (Mtwara)^[[25]](#footnote-25)^ | 95% (2015)^[[26]](#footnote-26)^ |  |
| **Quality Goal 4** | **x% reduction in the incidence of post-partum haemorrhage in health facilities** | | | | |
| **Indicator** | **Current performance (Uganda)** | | **Current performance (Tanzania)** | **Goal (Uganda)** | **Goal (Tanzania)** |
|  |  | |  |  |  |

| **Quality Goal 5** | **x% of all women who give birth commence breast-feeding within one hour of delivery** | | | | |
| --- | --- | --- | --- | --- | --- |
| **Indicator** | **Current performance (Uganda)** | | **Current performance (Tanzania)** | **Goal (Uganda)** | **Goal (Tanzania)** |
| Percentage of last children born in the five years preceding the survey were breastfed within 1 hour of birth  Percentage of last children born in the five years preceding the survey and who were ever breastfed, were breastfed within 1 hour of birth  ***Immediate breastfeeding*. Proportion of women with a live birth in last 24 months who put the newborn infant to the breast within 1 hour of birth** | 46.1%  45.4% (East Central)^[[27]](#footnote-27)^ | | 58%  64.8% (Mtwara)^[[28]](#footnote-28)^ | >70% (2015)^[[29]](#footnote-29)^ |  |
| **Quality Goal 6** | **x% of pre-term and low birth weight babies receive kangaroo mother care** | | | | |
| **Indicator (proposed (in bold))** | **Current performance (Uganda)** | **Current performance (Tanzania)** | | **Goal (Uganda)** | **Goal (Tanzania)** |
| **Proportion of women who gave birth to a LBW baby in the 24 months prior to survey who reported practicing KMC with their newborn** |  |  | |  |  |
| **Quality Goal 7** | **x% reduction in neonatal deaths due to asphyxia** | | | | |
| **Indicator** | **Current performance (Uganda)** | | **Current performance (Tanzania)** | **Goal (Uganda)** | **Goal (Tanzania)** |
|  |  | |  |  |  |
| **Quality Goal 8** | **x% reduction in incidence of maternal sepsis** | | | | |
| **Indicator** | **Current performance (Uganda)** | | **Current performance (Tanzania)** | **Goal (Uganda)** | **Goal (Tanzania)** |
|  |  | |  |  |  |
| **Quality Goal 9** | **x% reduction in incidence of neonatal sepsis** | | | | |
| **Indicator** | **Current performance (Uganda)** | | **Current performance (Tanzania)** | **Goal (Uganda)** | **Goal (Tanzania)** |
|  |  | |  |  |  |
| **Quality Goal 10** | **x% reduction in maternal deaths due to eclampsia/pre-eclampsia** | | | | |
| **Indicator** | **Current performance (Uganda)** | | **Current performance (Tanzania)** | **Goal (Uganda)** | **Goal (Tanzania)** |
|  |  | |  |  |  |

| **Quality Goal 11** | **x% women who gave birth in last month able to cite at least two danger signs in neonate** | | | |
| --- | --- | --- | --- | --- |
| **Indicator** | **Current performance (Uganda)** | **Current performance (Tanzania)** | **Goal (Uganda)** | **Goal (Tanzania)** |
| ***Knowledge of danger signs*. Proportion of women who had a live birth in the 24 months preceding survey who know at least 2 signs for seeking care immediately for their baby in the first month of life**  **(0‐28 days)** |  |  |  |  |
| **Quality Goal 12** | **x% women who gave birth in last month counselled on family planning** | | | |
| **Indicator** | **Current performance (Uganda)** | **Current performance (Tanzania)** | **Goal (Uganda)** | **Goal (Tanzania)** |
| Current use of modern contraceptives amongst married women aged 15-49  ***Contraceptive prevalence rate* The proportion of currently married/in union women aged 15‐49 who are using (or whose partner is using) a (modern or traditional) contraceptive method.** | 16.9% (East Central)^[[30]](#footnote-30)^ | 36.8% (Mtwara) ^[[31]](#footnote-31)^ |  |  |

*^1^ The wording of the indicator is according to the definition used in the health facility survey. However, the monitoring in the health facility will use ‘expected birth’ as the denominator and will use partly different time periods (calendar year or even shorter periods). Still also for monitoring in health facilities the same definition should be used.*

***Project Design***

EQUIP is an intervention study utilising a quasi experimental design to improve Maternal and Newborn Care through the use of Quality Improvement (QI) methods. The intervention focuses on improvements across the continuum of care for maternal and newborn health. Alongside the quality improvement activity, a parallel system will operate where data will be collected at all levels across the intervention and control districts through continuous surveys to produce a “report card” that is tailored to inform district health managers, health workers, and community members about issues in maternal and newborn health where they live and work.

In the two intervention districts, EQUIP will establish collaboratives at both community and health facility (HF) levels with respective QI teams connecting on the ground and overlapping at sub-district level with some sharing during learning sessions. The QI activity will be overseen and supported by middle and senior managers at district level with assistance from the in-country EQUIP personnel. District QI teams will work on barriers to achieving Collaboratives’ aims that fall outside of the influence or control of health facilities and their catchment communities.

**Uganda-Specific Design**

Every health facility (health centre or hospital) will form a QI team if one does not already exist. QI teams will be expected to meet at least once every two weeks and once a month these meetings will be supported by a quality mentor from the District. These monthly visits will continue for the first 12 months of the collaboratives’ programme and every two months thereafter.

Each parish will form a community QI team comprising two village health team members from each village within that parish. Most parishes cover approximately 8 villages. Whilst these volunteers are a recognised part of the formal healthcare infra-structure only approximately 25% are currently in post. Recruitment, based on the Ministry of Health’s selection criteria, will be an early task of the project. These village-based volunteers are already required to meet once a month at parish level under the direction of the Community Development Officer. QI activities will be added as a standing agenda item. Each HF QI team will be expected to liaise with the community QI teams that are working within its catchment area. One representative from each of these Community QI teams (usually two in total) will sit on the HF QI team whenever this is possible.

HF collaboratives will be formed at health sub-district level. Each health sub-district comprises between 11 to 17 health facilities. The collaborative participants will come together quarterly for learning sessions followed by action periods during which HF QI teams will test change ideas designed to improve performance around a specific part of the change package. They will work systematically through the continuum of care with new topic areas being introduced at each learning session. QI teams will continue to test change ideas in a given topic area until their performance is in line with the collaborative aims after which they will collect outcome data to ensure their improvement is sustained. Health facilities will be free to opt out of working in a specific change area if their baseline performance is in line with the collaborative aims.

The health facilities involved in the pilot will form part of the first collaborative to be launched. They will have more experience and knowledge of QI than other colleagues and will have already seen improvements in some of the areas covered in the change package. They will be encouraged to share their learning, continue to test changes that relate to their piloting activity and work on new topic areas introduced at the learning sessions.

Community collaboratives will also be formed at health sub-district level allowing for some overlap of learning session agendas where applicable. Each health sub-district covers approximately 23 parishes so 2 volunteers per community QI team could be accommodated at each learning session.

**Tanzania-Specific Design**

Each health facility (hospital, health centre or dispensary) will be asked to form a QI team if they don’t already exist. QI teams will be expected to meet at least once every two weeks and every month these meetings will be supported by a quality mentor from the District. This external support will continue for the first 12 months and then reduce to every other month.

Prior to the launch of the community collaborative, the project will work with communities to select two volunteers in each village to undertake EQUIP-related QI activity at community level. Community QI teams will be formed around the health facility governing committees which should operate at health facility level and comprise representatives from the community and HF. The committees are supposed to meet, at a minimum, every three months. In Tandahimba District they will be asked to meet monthly, include QI as part of their standing agenda and co-opt an EQUIP volunteer from each of the villages that fall within their catchment area. This will usually be 6-7 people, some of whom may already be board members. The health facility in-charge is the secretary to this committee. One of two QI mentors from the District will join the health facility governing committee meeting every month to support the QI work and mentor members of the group.

Health facility collaboratives will be formed at division level. Each division comprises between 8 to 15 health facilities. Representatives from the HF QI teams will come together quarterly for learning sessions followed by activity periods during which these teams will test change ideas designed to improve performance around a specific part of the change package. They will work systematically through the continuum of care with new topic areas being introduced at each learning session. As with the Uganda intervention, QI teams will test change ideas in a given topic area until their performance is in line with the collaborative aims after which they will continue to monitor their performance to ensure their improvement is sustained. Health facilities will be free to opt out of working in a specific change area if their baseline performance is in line with the collaborative aims.

The health facilities in Litehu Division have already been involved in the pilot phase of the project and as such are familiar with using some of the QI methodology and tools. These same facilities will transition easily from piloting to a fully-fledged collaborative with clear aims, measures and a change package that are agreed during the first learning session. They will be encouraged to continue testing those changes they selected for their pilot work while also focussing on new topic areas introduced at the learning sessions. Community collaboratives will also be formed at health sub-district level allowing for some overlap of learning session agendas where applicable. As community QI teams are formed at health facility level there will be 8 to 15 represented at each quarterly meeting.

**Time Lines**

The project is designed to assess the efficacy of QI as a means of reducing maternal and newborn mortality demonstrated through modelling techniques. In order for the impact of changes to be demonstrable at district level, the QI dosage needs to be as high as possible as quickly as possible. The piloting phase will therefore not exceed a maximum of 6 months even though it will not be possible to test ideas across the whole change package in this time. The sub-levels that include the pilot HFs will be the first collaboratives to launch followed 3 months later by the remaining two. This will allow some fast-tracking and learning from the pilots that should benefit the remainder of the Districts’ HFs and community QI teams. The QI intervention is due to conclude in year 4, quarter 2 of the project programme, allowing all of the collaboratives approximately 18 months to introduce changes

**Oversight**

In Uganda, the District Health Management Team and Council Health Management Team will oversee all the EQUIP project activity in the intervention district. This includes data collection, running the collaboratives and mentoring QI teams. The District QI Team will support the QI work at community and facility level and undertake its own improvements based on challenges that are encountered by HF and community teams. The District QI and management teams will be supported by the EQUIP in-country team and visited quarterly by the National QI Advisor

In Tanzania, the Council Health Management Team and the QI mentors will oversee the EQUIP project activity in the intervention district. This includes both the collaboratives and data collection. The District QI Team will support the QI work at community and facility level and undertake its own improvements based on challenges that are encountered by HF and community teams. The District QI team will be visited quarterly by the EQUIP team.

***Project Boundaries***

The project will operate across four districts; an intervention and comparison district in Tanzania (Tandahimba and Newala) and an intervention and comparison district in Uganda (Mayuge and Namaingo).

***Collaborative Structure***

**Project Team on the Ground**

In Uganda, the District has one quality management mentor who works on the DHMT’s behalf to ensure the EQUIP project is implemented [DN is this true?]. There is also a group of 4 from the DMHT who manage the HF QI teams and is responsible for their mentoring and coaching. In addition, 2 members of the DHMT manage the community QI teams along with parish-based Community Development Officers and Health Assistants who have also been trained in QI. These are charged with overseeing the community QI activities and mentoring the teams.

Tanzania has two QI mentors supporting the community QI teams and one mentor supporting the HF QI teams in addition to two in-country EQUIP experts [DN – this needs to be strengthened]

***Programme Sponsorship***

The project has an active dialogue with policymakers and practitioners at national and district level in the two countries, where a specific work-package is assigned to fill this role. Results will be reported back at district and national level and the project has received great interest from not the least Ministry of Health partners to inform national quality management interventions. National policymakers are fully briefed on the project, and may be called upon in case of need. At the international level a group of Scientific Advisors review and advise the project periodically, and give their input to the Project Steering Committee, which has monthly phone conferences. The Steering Committee reports back to the funder, the European Union.

***Initial Change Concepts***

The initial change package outlined below lists key interventions across the care continuum that if reliably executed are known to reduce maternal and neonatal mortality. The collaboratives will provide support to QI teams in identifying change ideas that they believe will enable these interventions to be performed. One output from the collaboratives will be a fully tested change package comprising change ideas that have led to demonstrable improvements in each of these intervention areas.

The change package for health centres has been divided into “basic” and “advanced” to reflect the differences in the range of services provided across these facilities. For example, health centres in Tanzania and level IV health centres in Uganda offer some of the same services available at the hospital while some dispensaries and level III and II health centres are not able to provide the full range of basic emergency obstetric care. It is not possible to anticipate what services any one health facility can offer from its categorisation alone (e.g. dispensary or health centre level III). The matching of the change package to facility will therefore need to take place through discussions with local HF QI teams and representatives from the DMHT at the start of the collaborative. It should be stressed that QI activity will enable many health facilities to expand the range of services they can offer but input limitations (equipment, supplies, staffing, utilities and buildings) may impose insurmountable constraints.

The number of interventions a QI team can successfully work on during any given action period is usually 1-2. Therefore over an 18 month period, collaborative teams are unlikely to be able to cover more than 9-12 topic areas in total. Interventions have therefore been prioritised based on the evidence for their impact in reducing maternal or neonatal mortality and the current performance in-country. Where HFs can demonstrate good results in high priority areas they will be invited to address topics given lower priority in the change package..

1. Ministry of Health and Social Welfare, United Republic of Tanzania, *The National Road Map Strategic Plan To Accelerate Reduction of Maternal, Newborn and Child Deaths in Tanzania 2008 – 2015* (2008) [↑](#footnote-ref-1)
2. Ministry of Health, Uganda, *Road Map for Accelerating the Reduction of Maternal and Neonatal Mortality and Morbidity in Uganda 2006-2015* (2007) [↑](#footnote-ref-2)
3. WHO and UNICEF, *Countdown to 2015 Decade Report (2000-2010). Taking stock of maternal, newborn and child survival*. (2010), WHO & UNICEF: Geneva. [↑](#footnote-ref-3)
4. UDHS 2006 (2007) [↑](#footnote-ref-4)
5. TDHS 2010 (2011) [↑](#footnote-ref-5)
6. Ministry of Health, Uganda, *Road Map for Accelerating the Reduction of Maternal and Neonatal Mortality and Morbidity in Uganda 2006-2015* (2007) [↑](#footnote-ref-6)
7. Ministry of Health and Social Welfare, United Republic of Tanzania, *The National Road Map Strategic Plan To Accelerate Reduction of Maternal, Newborn and Child Deaths in Tanzania 2008 – 2015* (2008) [↑](#footnote-ref-7)
8. UDHS 2006 (2007) [↑](#footnote-ref-8)
9. UDHS 2006 (2007) [↑](#footnote-ref-9)
10. TDHS 2010 (2011) [↑](#footnote-ref-10)
11. TDHS 2010 (2011) [↑](#footnote-ref-11)
12. Ministry of Health, Uganda, *Road Map for Accelerating the Reduction of Maternal and Neonatal Mortality and Morbidity in Uganda 2006-2015* (2007) [↑](#footnote-ref-12)
13. Ministry of Health and Social Welfare, United Republic of Tanzania, *The National Road Map Strategic Plan To Accelerate Reduction of Maternal, Newborn and Child Deaths in Tanzania 2008 – 2015* (2008) [↑](#footnote-ref-13)
14. National Bureau of Statistics (NBS) [Tanzania] and ICF Macro. *Tanzania Demographic and Health Survey 2010.* (2011) Dar es Salaam, Tanzania: NBS and ICF Macro. [↑](#footnote-ref-14)
15. UDHS 2006 (2007) [↑](#footnote-ref-15)
16. UDHS 2*006* (2007) [↑](#footnote-ref-16)
17. TDHS 2010 (2011) [↑](#footnote-ref-17)
18. Penfold S, Hill Z, Mrisho M, Manzi F, Tanner M, et al. (2010) *A Large Cross-Sectional Community-Based Study of Newborn Care Practices in Southern Tanzania.* PLoS ONE 5(12): e15593. doi:10.1371/journal.pone.0015593 [↑](#footnote-ref-18)
19. Penfold et al (2010) [↑](#footnote-ref-19)
20. Penfold et al (2010) [↑](#footnote-ref-20)
21. Penfold et al (2010) [↑](#footnote-ref-21)
22. Penfold et al (2010) [↑](#footnote-ref-22)
23. Ministry of Health, Uganda, *Road Map for Accelerating the Reduction of Maternal and Neonatal Mortality and Morbidity in Uganda 2006-2015* (2007) [↑](#footnote-ref-23)
24. Ministry of Health, Uganda, *Road Map for Accelerating the Reduction of Maternal and Neonatal Mortality and Morbidity in Uganda 2006-2015* (2007) [↑](#footnote-ref-24)
25. TDHS 2010 (2011) [↑](#footnote-ref-25)
26. Ministry of Health, Uganda, *Road Map for Accelerating the Reduction of Maternal and Neonatal Mortality and Morbidity in Uganda 2006-2015* (2007) [↑](#footnote-ref-26)
27. UDHS *2006*  (2007) [↑](#footnote-ref-27)
28. TDHS *2010* (2011) [↑](#footnote-ref-28)
29. Ministry of Health, Uganda, *Road Map for Accelerating the Reduction of Maternal and Neonatal Mortality and Morbidity in Uganda 2006-2015* (2007) [↑](#footnote-ref-29)
30. UDHS 2006 (2007) [↑](#footnote-ref-30)
31. TDHS *2010* (2011) [↑](#footnote-ref-31)
