# Supplementary material for: Effects of the EQUIP quasi-experimental study testing a collaborative quality improvement approach for maternal and newborn health care in Tanzania and Uganda
Source: Implement Sci. 2017 Jul 18;12:89. doi: 10.1186/s13012-017-0604-x (PMC5516352; doi:10.1186/s13012-017-0604-x)
Supplement: Additional file 1: — Webannex I EQUIP Maps. Webannex II EQUIP mentoring and coaching. Webannex III EQUIP Timeline of assessment and implementation. Webannex IV Project charter. Webannex V EQUIP Example report card. Webannex VI Vignettes. Webannex VII EQUIP Example Runchart. Webannex VIII EQUIP Example Analysis. (ZIP 1064.96 kb) [file 13012_2017_604_MOESM1_ESM.zip › Webannex V EQUIP Example report card.docx]

**Webannex 5: Report card displaying results from household surveys**

**
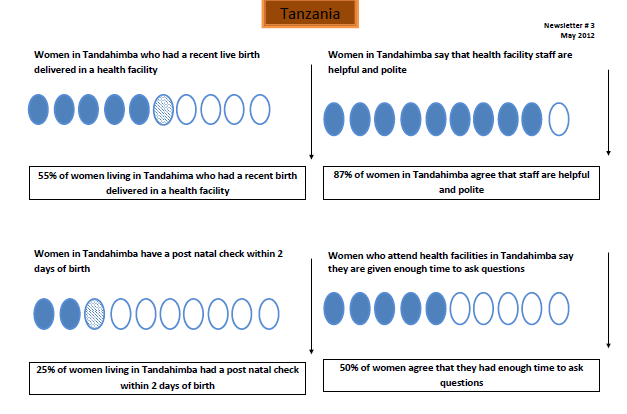
**

Figure: This community report card graphically displayed the proportion of mothers who reported (1) having delivered in a facility (2) found the health worker helpful ( 3) had a postnatal check-up and (4) were given enough time to ask questions. The English translation from the original Kswahilli version shared with community members is shown.
