# Supplementary material for: Effects of the EQUIP quasi-experimental study testing a collaborative quality improvement approach for maternal and newborn health care in Tanzania and Uganda
Source: Implement Sci. 2017 Jul 18;12:89. doi: 10.1186/s13012-017-0604-x (PMC5516352; doi:10.1186/s13012-017-0604-x)
Supplement: Additional file 1: — Webannex I EQUIP Maps. Webannex II EQUIP mentoring and coaching. Webannex III EQUIP Timeline of assessment and implementation. Webannex IV Project charter. Webannex V EQUIP Example report card. Webannex VI Vignettes. Webannex VII EQUIP Example Runchart. Webannex VIII EQUIP Example Analysis. (ZIP 1064.96 kb) [file 13012_2017_604_MOESM1_ESM.zip › Webannex VII EQUIP Example Runchart.docx]

**Webannex 7: Example of run chart following facility deliveries**

**
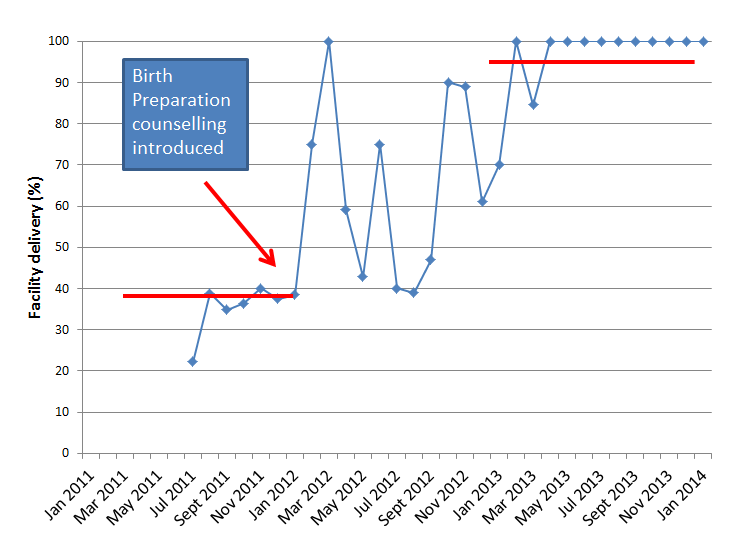
**

Figure: Run chart indicating the proportion of facility deliveries compared to registered home and facility birth over the period July 2011 to January 2014 from one health centre. The red lines indicate the average before and after the intervention.

**Webannex 6b:**

Figure 3: Trend of facility delivery compiled from all health facilities in intervention district (denominator is reported facility and home deliveries)

(Explanation: The run-chart of all recorded deliveries (home and facility deliveries) suggest a similar trend of increased proportion of facility delivery per all recorded births as obtained through the continuous household survey (see webannex 6). However, the coverage rates are higher than our population based data indicate. A likely explanation is that home deliveries are probably not sufficiently captured in the facility-based health management information system.
