# Supplementary material for: Effects of the EQUIP quasi-experimental study testing a collaborative quality improvement approach for maternal and newborn health care in Tanzania and Uganda
Source: Implement Sci. 2017 Jul 18;12:89. doi: 10.1186/s13012-017-0604-x (PMC5516352; doi:10.1186/s13012-017-0604-x)
Supplement: Additional file 1: — Webannex I EQUIP Maps. Webannex II EQUIP mentoring and coaching. Webannex III EQUIP Timeline of assessment and implementation. Webannex IV Project charter. Webannex V EQUIP Example report card. Webannex VI Vignettes. Webannex VII EQUIP Example Runchart. Webannex VIII EQUIP Example Analysis. (ZIP 1064.96 kb) [file 13012_2017_604_MOESM1_ESM.zip › Webannex VIII EQUIP Analysis Example.docx]

**Webannex 8: Example graphs illustrating the statistical analysis of impact, example of Skilled attendant at delivery (Percentage of Women aged 13-49 yrs reporting a skilled attendant at their most recent delivery (within past 12 months)) – Tanzania**

In the following two graphs we illustrate the method used to estimate the impact of EQUIP on skilled attendance at delivery in intervention (Tandahimba) versus comparison (Newala) districts in Tanzania. We used an approach adapted from the analytic methods often used for interrupted time-series. For each of the six time points of the continuous survey rounds, we calculated the prevalence of skilled attendance at delivery (graph a) and the difference prevalence between the intervention and comparison districts (graph b). We used meta-regression to fit a regression line through the resulting six data points over the 30 months of data collection (graph b) and used this regression to estimate the difference-of-differences value between intervention and comparison districts from baseline (first data collection round) to endline (last data collection round). In this example, the modelled difference between intervention and comparison districts at baseline was -2 percentage points, and that at endline was 9 percentage points (graph b), giving a difference-of-differences value between intervention and comparison districts from baseline to endline of 11 percentage points (CI -3, 25). Thus the reported percentage points increase does not represent the crude differences between 1^st^ and 6^th^ round of data collection but use the modelled differences. The delta method was used to estimate the variance of the difference of differences measure and present confidence intervals.


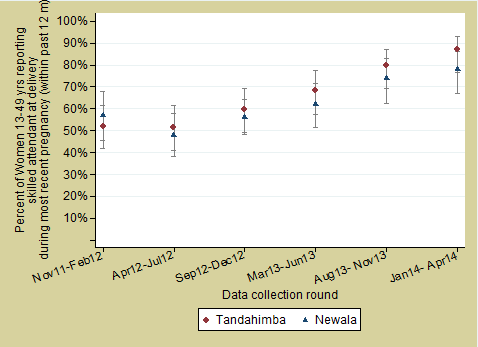


**Figure a**: Percentage of Women aged 13-49 yrs reporting a skilled attendant at their most recent delivery (within past 12 months) in Tandahimba and Newala districts, Tanzania


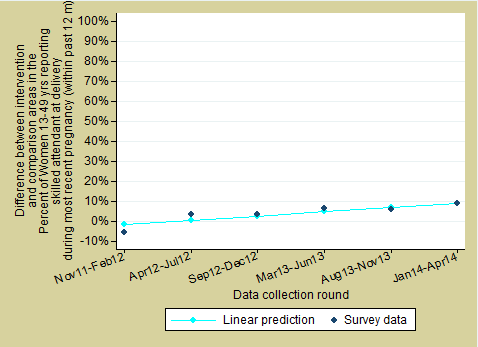


Figure b: Absolute and modelled differences between intervention and comparison districts in the percentage of Women aged 13-49 yrs reporting a skilled attendant at their most recent delivery (within past 12 months), Tanzania
