# Supplementary material for: Effects of the EQUIP quasi-experimental study testing a collaborative quality improvement approach for maternal and newborn health care in Tanzania and Uganda
Source: Implement Sci. 2017 Jul 18;12:89. doi: 10.1186/s13012-017-0604-x (PMC5516352; doi:10.1186/s13012-017-0604-x)
Supplement: Additional file 1: — Webannex I EQUIP Maps. Webannex II EQUIP mentoring and coaching. Webannex III EQUIP Timeline of assessment and implementation. Webannex IV Project charter. Webannex V EQUIP Example report card. Webannex VI Vignettes. Webannex VII EQUIP Example Runchart. Webannex VIII EQUIP Example Analysis. (ZIP 1064.96 kb) [file 13012_2017_604_MOESM1_ESM.zip › Webannex VI Vignettes.doc]

**Webannnex 6: Vignettes**

**Health Facility Vignettes: Uganda**

*One thing that was emphasized during mentoring and coaching at the health facilities was the active management of third stage of labour. A very important change idea created by quality improvement teams was to ensure that all of the items for labour were ready—especially oxytocin—on the tray beside the woman during labour. To minimize stock-outs, health facility staff also request for oxytocin in bulk from the national medical stores based on the number of deliveries that they have in a month. A problem at lower-level health facilities is that they cannot order in bulk like higher-level facilities can, so to ensure a strong network of healthcare provision within a certain area, staff at higher-level facilities order oxytocin in bulk, not only for their facility, but for lower-level facilities as well. They want to maintain the networks of support. This was not done before EQUIP. Before EQUIP, there were often stock-outs of oxytocin, and its administration was lower. If women could not be helped, they were referred onwards—it’s true, we lost mothers’ lives. –Rogers Mandu, EQUIP staff Uganda*

**Birth Preparedness**

*Following the introduction of EQUIP, a change idea used at many health facilities was to employ “goal-oriented antenatal care”, whereby what is supposed to happen on the first visit, second visit, third visit, and so on is shared with the mothers, and dates for future appointments are fixed. Because of this change idea, there has been a large increase in regular antenatal care attendance. There are now special health education sessions during antenatal care, and within those sessions, it is checked whether the woman has made all of the correct birth preparations. Volunteers also check these preparations in the community. Previously women were not attending antenatal care as often and therefore not receiving this education. Due to the success of this change idea, women are more actively making birth preparations. –Rogers Mandu, EQUIP staff Uganda*

**Active Management of the Third Stage of Labour**

*Prior to EQUIP, there were three problems: many health facility staff could not use a partograph to monitor labour; many staff simply did not use a partograph; and partographs were not available. Change ideas were introduced in response to these problem. At learning sessions, there has been continuous education for anyone responsible for conducting deliveries so that they are aware of all of the stages of active management of the third stage of labour, including the use of the partograph. Many photocopies of partographs were made and distributed. Reminders to use partographs were also put up on the walls of the labour ward. A file was created to store the partographs to be checked to ensure that they were used for every mother and that they were filled in correctly. The maternity ward in-charges as well as the quality improvement mentors will check these files. These change ideas are now used in almost every facility, because they have been successful and health facility staff learn from one another’s best practices. We have seen a great improvement. –Rogers Mandu, EQUIP staff Uganda*

**Postnatal Care**

*One fundamental change about postnatal care in the facilities is that the postnatal care should be given within the first six days, because previously they were doing postnatal care within the first six weeks after delivery. Recognizing this as a major problem, the shorter period was introduced by the quality improvement teams, with visits within the first six hours, then the first six days, then the first six weeks. It is called the “666 element”. All of the health facilities have started introducing the new strategy. Now most babies and mothers are seen within the first six days. For each of the health facility team, they have a documentation journal for each of the quality improvement indicators that they are monitoring, such as the percentage of babies seen within six days after birth. The mentors are then able to review their documentation to make sure that the visits were conducted. There has definitely been an improvement. There are so many reports about babies that have been saved when they had difficulties shortly after delivery. There is also emphasis on the community about going to the facility and reporting early to the facility. So many mothers have been saved. –Rogers Mandu, EQUIP staff Uganda*

**Measuring Blood Pressure**

*There was a big problem with women not having their blood pressure taken during antenatal care. By using PDSA cycles, it was discovered that blood pressure was often taken but not recorded, or not taken at all, often because the machines were not good. Three main change ideas were introduced in almost all facilities: to add a column in the antenatal care books for blood pressure; to put up signs in the health facility to remind staff to take blood pressure; and to put in requisitions for new machines. Many women with pre-eclampsia and eclampsia have had it detected and have been helped as a result. We’ve also seen mothers being referred from lower level facilities, which is specifically a result of regular taking of blood pressure measurements. –Rogers Mandu, EQUIP staff Uganda*

**Weighing Mothers and Babies**

*Mothers and newborns were not regularly weighed until PDSA cycles around this topic began. After newborns began to be regularly weighed as part of a change idea, health facility staff discovered that there was a gap in the management of the low birthweight babies who were previously not detected. As a result of regular weighing of newborns, kangaroo mother care was introduced. –Rogers Mandu, EQUIP staff Uganda*

**Generally**

*Another significant change is that the culture of documentation has improved significantly throughout the intervention district in health facilities. They are documenting the procedures, the results, and what the actions taken were. This has improved across the board. It hasn’t happened necessarily as a result of a change idea, but it is something that is happening as a result of EQUIP. During each mentoring visit, performance is reviewed, so teams are consciously doing documentation because they are aware that their work will be reviewed. Some of the staff who don’t even do quality improvement have recognised the importance of documentation. Documentation journals have been started even for areas not targeted by EQUIP. The impact of quality improvement has helped more broadly across these facilities. –Rogers Mandu, EQUIP staff Uganda*

**Community Vignettes: Uganda**

**Generally**

*For the first time, the community is collecting data and using it for improvement. They collect this information, the number of households, the number of pregnant women, and so on, and then they will send it to someone else, either at the county or district level. For the first time the community is collecting data for decision-making. The community now knows why they are collecting data and how they can use it. Because they are collecting data, community volunteers have improved their mechanism of monitoring and follow up. The cohesion within the groups has also improved. For the case of the quality improvement teams, the sharing of data has motivated them to meet monthly so that they can reflect on the data. To the largest extent, most of the volunteers are empowered by the data that they collect themselves. Over 85% are very, very excited about collecting and using data. –Rogers Mandu, EQUIP staff Uganda*

*As a result of quality improvement activities, community volunteers have begun to understand some of the bottlenecks and challenges that are hindering mothers attending antenatal care and receiving postnatal care. Volunteers came up with the change idea that on some of the busier days at the health facilities, they can help the health facility staff with the things that are not as technical, like weighing. This change idea is widespread throughout the district, especially in the villages that were early adopters of the intervention, as they started to use it and then peer learning allowed for the spread of this change idea. Now we have a number of mothers and newborns being followed up, especially for busy things like antenatal care and also postnatal care. In some of the health facilities, the volunteers were educating the mothers on postnatal care in the community. A mother who has delivered is followed up in the community, given a referral, and goes back to the health facility. –Rogers Mandu, EQUIP staff Uganda*

**Birth Preparedness and Health Facility Delivery**

*Initially, when we did orientation and training of the volunteers and the quality improvement teams at the community level, we taught them the steps that we go through: the problem analysis and brainstorming and developing possible solutions. Some of the volunteers suggested the change idea that money needed to be saved to assist the mothers in the community to go to the nearest health facility. Women’s savings groups were then started. During home visits, if volunteers noticed a woman was struggling to prepare for birth, she would be told about the savings groups. Information was also given to all pregnant women in a village during a special meeting. Now there are over 45 of these groups. They work together to save small amounts throughout each woman’s pregnancy. If this is done over a period, there could be significant amounts of money available. Women have expressed their joy and happiness about how they have been assisted. There was one mother who was brought to the health centre to deliver, but the midwife wasn’t there. She didn’t have enough money to get to another health facility, so she used the money from the savings groups to go to a higher-level facility and delivered safely. In general, many women are able to use the money from the savings groups to prepare for birth. Before, if a woman could not prepare for birth, she was afraid to go to the health facility and would instead give birth at home. The women are now comfortably and confidently going to the facility to deliver. –Rogers Mandu, EQUIP staff Uganda*

**Antenatal Care Attendance**

*There was emphasis during antenatal care that women had to come with their husbands to be tested for HIV. The EQUIP volunteers provided a lot of sensitization, explaining that if HIV is detected, you can prevent the transmission to the child. The volunteers make a point of going to houses when the husband is available. So a change idea to provide education during home visits to couples, not just the woman, was created. Especially in areas that are neighbouring the forests and the fishing communities, the men will spend a lot of time away from their homes, so volunteers target their visits at the time when the men are likely to be at their homes. Generally this change idea is widespread. The men are getting more involved. We now have stories about husbands supporting their wives in doing birth preparedness, they are escorting their wives to antenatal care, and they ensure women deliver in the health facility. –Rogers Mandu, EQUIP staff, Uganda*

**Immunization of Newborns**

*It was difficult for babies to get all of their essential immunizations and to complete the full doses. Community volunteers realized the problem was that sometimes the women find it hard to move long distances with small babies. Especially in very hard-to-reach areas like the forest areas and along the shores, volunteers would mobilize the mothers for an outreach immunization service. On the day that they mobilize women and when they know the vaccines are available, they communicate the time and location to a health worker, who then provides the immunizations to the babies. Another problem identified by volunteers was that in lower-level facilities, they lack a refrigerator and cannot maintain the cold chain. Therefore, a change idea created was to bring vaccines from a nearby health facility that does have a refrigerator. As a result, there has been an improvement in immunization coverage. –Rogers Mandu, EQUIP staff Uganda*

**Health Facility Vignettes: Tanzania**

**Community Vignettes: Tanzania**

**General**

*Volunteers in one cluster in particular would often mention that when women are in labour, they feel that they will get better services at the health facility because they were showing up with the volunteers. This is an indication that the women respect the volunteers and that they have made a link between the volunteers and health facility delivery. After receiving education, mothers know that there is a good relationship between the volunteers and the health facility staff. It shows that the implementation has increased trust among members of the community towards the volunteers. –Albert Majura, EQUIP Community Quality Improvement Coordinator Tanzania*

**Health Facility Delivery**

*In multiple villages, volunteers are being provided with an annual allowance from funds from the village cashew sales directly. This allowance is important because it shows that leaders in the village and the community at large see the importance of quality improvement. They see the work that is being done by the volunteers in reducing maternal and newborn health problems. They want to recognize the good work of the volunteers to motivate them. –Albert Majura, EQUIP Community Quality Improvement Coordinator Tanzania*

*In one cluster, a staff member from a nearby health facility rushed to be present for a cluster meeting. She wanted to show the community volunteers that there had been an increase in home births recently, and she wanted to encourage them to increase their efforts. Nowadays, even those working in the health facilities are seeing the importance of volunteers and are recognizing that they can make a difference in maternal and newborn health. They can help each other in the community to make sure that there is no maternal and newborn death. Now health facility staff are cooperating at the community level and seeing it as necessary in order to encourage good health-seeking behaviours. –Albert Majura, EQUIP Community Quality Improvement Coordinator Tanzania*

*Volunteers in one community recognized that it was a struggle for women to get to the health facility for delivery when her husband was not present. Part of the problem was a lack of funds when a male was not around. In this village, volunteers had a change idea to create a village emergency fund that should be repaid once a husband returned. The community supported this idea because they have seen the benefit of a mother delivering in a health facility and they are trying to find ways to make it easier for mothers to get to the health facility. –Albert Majura, EQUIP Community Quality Improvement Coordinator Tanzania*

**Birth Preparedness**

*One time the Minister of Health said that “every woman should come with her husband to antenatal care”, but there was no success and we saw very few men attending. They did not have the knowledge about the health of their wives; they were just staying at home. It has been a long-time thing to try to encourage male involvement, but people didn’t comply. We realized that it was very important to engage with men, because when volunteers were using PDSA cycles to test change ideas around providing health education at homes to encourage women to deliver in the health facility, mothers were not abiding to the education. Other times, husbands became very harsh because they didn’t understand why the volunteers were visiting. So, the volunteers sat down and decided that they needed to adapt their change idea to involve men when providing education.* *Now in Tandahimba, because the EQUIP volunteers and health facility staff encourage male involvement, men are moving. They go with their wives to antenatal care and they are told about birth preparedness, the danger signs, and everything that might happen during the pregnancy. It has taught them to take care, because they know that if their wife delivers at home she can lose her life. This is why, even if a wife fails to remember, the husband will remind her about her appointments. Now that men are getting more involved, positive maternal and newborn health behaviours are being encouraged by men within their own homes. In other districts they are not doing as they are doing in Tandahimba. –Albert Majura, EQUIP Community Quality Improvement Coordinator Tanzania*
